# Evaluating Sentinel-5P TROPOMI tropospheric NO_2_ column densities with airborne and Pandora spectrometers near New York City and Long Island Sound

**DOI:** 10.5194/amt-13-6113-2020

**Published:** 2020-11-17

**Authors:** Laura M. Judd, Jassim A. Al-Saadi, James J. Szykman, Lukas C. Valin, Scott J. Janz, Matthew G. Kowalewski, Henk J. Eskes, J. Pepijn Veefkind, Alexander Cede, Moritz Mueller, Manuel Gebetsberger, Robert Swap, R. Bradley Pierce, Caroline R. Nowlan, Gonzalo González Abad, Amin Nehrir, David Williams

**Affiliations:** 1 NASA Langley Research Center, Hampton, VA 23681, USA; 2 Office of Research and Development, United States Environmental Protection Agency, Triangle Research Park, NC 27709, USA; 3 NASA Goddard Space Flight Center, Greenbelt, MD 20771, USA; 4 Universities Space Research Association, Columbia, MD 21046, USA; 5 Royal Netherlands Meteorological Institute (KNMI), De Bilt, the Netherlands; 6 Department of Geoscience and Remote Sensing, Delft University of Technology, Delft, the Netherlands; 7 LuftBlick, Kreith, Austria; 8 University of Wisconsin–Madison Space Science and Engineering Center, Madison, WI 53706, USA; 9 Harvard-Smithsonian Center for Astrophysics Cambridge, MA 02138, USA

## Abstract

Airborne and ground-based Pandora spectrometer NO_2_ column measurements were collected during the 2018 Long Island Sound Tropospheric Ozone Study (LISTOS) in the New York City/Long Island Sound region, which coincided with early observations from the Sentinel-5P TROPOspheric Monitoring Instrument (TROPOMI) instrument. Both airborne- and ground-based measurements are used to evaluate the TROPOMI NO_2_ Tropospheric Vertical Column (TrVC) product v1.2 in this region, which has high spatial and temporal heterogeneity in NO_2_. First, airborne and Pandora TrVCs are compared to evaluate the uncertainty of the airborne TrVC and establish the spatial representativeness of the Pandora observations. The 171 coincidences between Pandora and airborne TrVCs are found to be highly correlated (*r*^2^ =0.92 and slope of 1.03), with the largest individual differences being associated with high temporal and/or spatial variability. These reference measurements (Pandora and airborne) are complementary with respect to temporal coverage and spatial representativity. Pandora spectrometers can provide continuous long-term measurements but may lack areal representativity when operated in direct-sun mode. Airborne spectrometers are typically only deployed for short periods of time, but their observations are more spatially representative of the satellite measurements with the added capability of retrieving at subpixel resolutions of 250m×250m over the entire TROPOMI pixels they overfly. Thus, airborne data are more correlated with TROPOMI measurements (*r*^2^ = 0.96) than Pandora measurements are with TROPOMI (*r*^2^ = 0.84). The largest outliers between TROPOMI and the reference measurements appear to stem from too spatially coarse a priori surface reflectivity (0.5°) over bright urban scenes. In this work, this results during cloud-free scenes that, at times, are affected by errors in the TROPOMI cloud pressure retrieval impacting the calculation of tropospheric air mass factors. This factor causes a high bias in TROPOMI TrVCs of 4%–11%. Excluding these cloud-impacted points, TROPOMI has an overall low bias of 19%–33% during the LISTOS timeframe of June–September 2018. Part of this low bias is caused by coarse a priori profile input from the TM5-MP model; replacing these profiles with those from a 12 km North American Model–Community Multiscale Air Quality (NAMCMAQ) analysis results in a 12%–14% increase in the TrVCs. Even with this improvement, the TROPOMI-NAMCMAQ TrVCs have a 7%–19% low bias, indicating needed improvement in a priori assumptions in the air mass factor calculation. Future work should explore additional impacts of a priori inputs to further assess the remaining low biases in TROPOMI using these datasets.

## 1 Introduction

Nitrogen dioxide (NO_2_) is an air pollutant emitted naturally through soil emissions and lightning, as well as anthropogenically as a combustion product from sources such as mobile vehicles, powerplants, and industrial processes. NO_2_ is harmful to human health (e.g., Fischer et al., 2015; Anenberg et al., 2018) both directly and through its role in the production of near-surface ozone and particulate matter, making it a criteria air pollutant monitored and regulated by the Clean Air Act (https://www.epa.gov/clean-air-act-overview: last access: 18 April 2020). Due to its short lifetime of a few hours as a component of NO_*x*_ (NO+NO_2_) (Liang et al., 1998; Beirle et al., 2011; Liu et al., 2016), the spatial distribution of NO_2_ near anthropogenic emission sources is highly heterogeneous, with complex patterns that are hard to characterize from sparse networks of ground-based monitors.

The TROPOspheric Monitoring Instrument (TROPOMI) on board the Copernicus Sentinel-5 Precursor (S5P) satellite currently measures column densities of NO_2_ globally at unprecedented spatial resolution, making it an important tool for studying and monitoring urban air pollution. TROPOMI continues a long legacy of ultraviolet–visible (UV–VIS) backscatter measurements from satellites observing trace gas column densities related to air quality (González Abad et al., 2019). Global NO_2_ measurements have heritage from the Global Ozone Monitoring Experiment (GOME; Burrows et al., 1999), SCanning Imaging Absorption spectroMeter for Atmospheric CHartographY (SCIAMACHY; Bovensmann et al., 1999), GOME-2 (Callies et al., 2000; Behrens et al., 2018), Ozone Monitoring Instrument (OMI; Levelt et al., 2006; Levelt et al., 2018), Ozone Mapping and Profiling Suite (OMPS; Yang et al., 2014), and as of October 2017, TROPOMI (Veefkind et al., 2012) aboard S5P. Over the last couple decades, the spatial and temporal resolution of these satellite NO_2_ products have improved, with the first daily global coverage achieved by OMI launched in 2004 and with TROPOMI achieving a spatial resolution an order of magnitude finer (currently approximately 3.5 km×5.5 km at nadir) than the still-operating OMI (13 km×24 km at nadir) and OMPS (50 km×50 km at nadir on Suomi NPP) instruments.

The use of the TROPOMI tropospheric NO_2_ products for applications such as evaluating emissions inventories and distinguishing point sources has already been documented in recent literature. Goldberg et al. (2019) used data from the first year of TROPOMI operation to evaluate top-down NO_*x*_ emissions over three major US cities and two large powerplants. Complementary studies also pinpointed emissions from large point sources (Beirle et al., 2019) and even showed that emissions in Paris, France, have not decreased as expected since 2012 (Lorente et al., 2019). Griffin et al. (2019) found that the improved spatial resolution of TROPOMI was able to distinguish NO_2_ plumes from individual sources near the Canadian Oil Sands, which was not possible with the coarser measurements from OMI.

To enhance the integrity of using TROPOMI data in research and applications, each product requires systematic evaluation and validation. Validation activities include evaluating the data products under polluted and clean scenes using reference measurements from satellite, airborne, and ground-based instrumentation (van Geffen et al., 2019). Routine TROPOMI NO_2_ validation reports are produced regularly and documented at http://mpc-vdaf.tropomi.eu/ (last access: 30 March 2020). Additional in-depth studies in recent literature have been mostly confined to ground-based column measurements from multiaxis differential optical absorption spectroscopy (MAX-DOAS) and/or direct-sun column measurements (e.g., from Pandora spectrometers) (e.g., Griffin et al., 2019; Zhao et al., 2020; Ialongo et al., 2020, Wang et al., 2020). These types of measurements have been used in the past to evaluate the OMI Tropospheric Vertical Column (TrVC) product, though this was shown to be challenging in polluted areas as spatial variability in NO_2_ can result in sampling mismatches between the small spatial scale measurements from the ground-based spectrometers and the >300 km^2^ pixels from OMI (Lamsal et al., 2014; Reed et al., 2015; Goldberg et al., 2017; Judd et al., 2019). Initial results of TROPOMI NO_2_ product validation with Pandora spectrometer direct-sun measurements show more encouraging results with higher levels of correlation than OMI evaluations (OMI examples found in Goldberg et al., 2017, and Judd et al., 2019; TROPOMI examples found in Griffin et al., 2019, Zhao et al., 2020, Ialongo et al., 2020, and this work).

In addition to ground-based column measurements, airborne column mapping datasets have been identified as valuable for TROPOMI TrVC validation efforts (van Geffen et al., 2019). Airborne spectrometers have the capability to map at much finer spatial resolutions than current satellite-based observations; for example, those used in this study have a spatial resolution of approximately 250m×250 m. Airborne spectrometers have been used to visualize high spatiotemporal variations in NO_2_ over select areas in Europe, North America, Africa, and Asia (Popp et al., 2012; Schönhardt et al., 2015; Lawrence et al., 2015; Nowlan et al., 2016, 2018; Lamsal et al., 2017; Meier et al., 2017; Tack et al., 2017, 2019, Broccardo et al., 2018; Judd et al., 2018, 2019) and have even contributed toward evaluating emissions inventories and ozone production sensitivity (Schönhardt et al., 2015; Souri et al., 2018; Souri et al., 2020). Measurements from airborne spectrometers have also been compared to the OMI NO_2_ products. Broccardo et al. (2018) found that agreement between the airborne mapper, iDOAS, and OMI improves with distance away from large emission source regions. Lamsal et al. (2017) discovered moderate correlation during a small subset of comparisons between the Airborne Compact Atmospheric Mapper (ACAM) and OMI over the Maryland region in 2011, though large differences were found for instances with insufficient sampling by the airborne mapper in areas subject to spatial heterogeneity of NO_2_. The large pixels from OMI are difficult to completely sample with airborne spectrometer observations; however, with the improved spatial resolution of TROPOMI, undersampling by airborne spectrometers is less of a concern though it can still impact statistical analysis between airborne spectrometers and TROPOMI as was demonstrated by Tack et al. (2020) as well as the work presented in this paper.

In this study, we use data from two NASA airborne spectrometers and nine ground-based (Pandora) spectrometers to evaluate the S5P TROPOMI NO_2_ TrVC v1.2 product over New York City (NYC) and Long Island Sound during the summer 2018 Long Island Sound Tropospheric Ozone Study (LISTOS) field campaign. The intercomparisons between the three independent datasets help bound NO_2_ product uncertainties due to spatial and temporal variability and a priori assumptions within the retrievals. Section 2 introduces LISTOS and each NO_2_ dataset: S5P TROPOMI, the airborne spectrometers, and Pandora spectrometer, along with details on methodology. Section 3 evaluates the airborne spectrometer retrieval using Pandora measurements. Section 4 presents comparisons of TROPOMI NO_2_ columns to the airborne spectrometer observations during LISTOS. Section 5 compares TROPOMI NO_2_ TrVCs to Pandora spectrometer data for the LISTOS timeframe as well as expanded through winter 2019. Throughout these sections causes for bias in the TROPOMI product based on the a priori profile and cloud assumptions are discussed. Section 6 summarizes TROPOMI NO_2_ TrVC performance in the NYC region, and Sect. 7 presents concluding remarks. Together these results demonstrate TROPOMI’s capability for observing the spatial distribution of NO_2_ in heterogeneous environments and demonstrate approaches for resolving apparent differences associated with linking observations from different measurement strategies.

## 2 Data and methods

### 2.1 The Long Island Sound Tropospheric Ozone Study

Data in this study were acquired across the NYC and Long Island Sound region in the United States as part of the Long Island Sound Tropospheric Ozone Study (LISTOS: https://www.nescaum.org/documents/listos; https://www-air.larc.nasa.gov/missions/listos/index.html: last access: 18 April 2020). LISTOS was a multiorganizational collaborative air quality study focused on understanding the sources and temporal emission profiles of the ozone precursors, nitrogen oxides (NO_*x*_) and volatile organic compounds (VOCs), across the NYC metropolitan area and ozone formation and transport in this coastal region. Measurements conducted include in situ and remotely sensed air quality and meteorology measurements from satellites, aircraft, and ground sites as well as the integration of the measurements with air quality models. This urban to suburban coastal area is a diverse region for validating satellite products due to the heterogeneous patterns in pollution as well as varying environmental factors such as surface reflectivity. In this study, we consider measurements from the LISTOS timeframe to span late June through September 2018, though some measurements extended before and after this time period.

### 2.2 S5P TROPOMI

Sentinel-5 Precursor (S5P) was launched October 2017 into a sun-synchronous low Earth orbit with a 13:30 local Equator crossing time. S5P carries a single instrument, TROPOMI, which consists of a hyperspectral spectrometer observing eight bands spanning the ultraviolet (UV), visible (VIS), near-infrared, and shortwave infrared portions of the electromagnetic spectrum (Veefkind et al., 2012). The S5P orbit combined with the wide TROPOMI swath width of 2600 km provides observations between approximately 17:00 and 19:00 UTC (13:00–15:00 EDT) over the New York City and Long Island Sound region, capturing the early afternoon spatial distribution of trace gas columns including CO (Borsdorff et al., 2018), HCHO (De Smedt et al., 2018), CH_4_ (Hu et al., 2018), NO_2_ (van Geffen et al., 2019, 2020), SO_2_ (Theys et al., 2017), and O_3_ (Garane et al., 2019).

In this work, the TROPOMI v1.2 NO_2_ TrVC product is evaluated with airborne and ground-based column density measurements from 25 June 2018 to 19 March 2019 over the LISTOS domain. The retrieval is built on the heritage of the Ozone Monitoring Instrument DOMINO product (Boersma et al., 2011), including developments from the QA4ECV project (Boersma et al., 2018; van Geffen et al., 2019; http://www.qa4ecv.eu/: last access: 18 April 2020). NO_2_ total slant columns are retrieved via the differential optical absorption spectroscopy (DOAS; Platt and Stutz, 2008) method in the visible window of 405–465 nm. Following the spectral fit, the slant columns are separated into their stratospheric and tropospheric components. The stratospheric column is estimated by assimilating the total columns in the TM5-MP model. The remaining tropospheric slant columns are converted into vertical columns through the calculation and application of air mass factors (AMFs; Palmer et al., 2001). A priori inputs for the tropospheric NO_2_ AMF calculations include viewing and solar geometry, surface pressure, and NO_2_ profile shape from the 1° × 1° TM5-MP model (Williams et al., 2017), 0.5° × 0.5° surface albedo climatology built upon 5 years of OMI data (Kleipool et al. 2008), and the FRESCO-S cloud fraction and cloud height (Loyola et al., 2018) (Table 1).

TROPOMI data during the time period of this analysis have a nadir spatial resolution of 3.5 km×7 km, with pixel areas ranging from 32.5 to 129.5 km^2^. Beginning on 6 August 2019, the nadir spatial resolution of the TROPOMI NO_2_ product is refined to 3.5 km×5.5 km (Ludewig et al., 2020). TROPOMI is capable of observing pollution at a spatial resolution a factor of 10 times more refined than its predecessor satellite sensor, OMI (Levelt et al., 2006, 2018).

Only TROPOMI data with qa_value=1 are considered in this analysis, which removes pixels influenced by issues such as sun glint, missing retrieval information, or cloud radiative fractions (CRFs) above 50% (van Geffen et al., 2019, Eskes et al., 2019). We note that qa_values down to 0.75 are deemed acceptable for most data uses, but 2% or less of the TROPOMI data in this work had qa_values between 0.75 and 1 and do not affect the results. This work also makes use of the averaging kernel and pressure profiles used in the retrieval to explore the impact of different NO_2_ profile shapes within the air mass factor calculation and explores sensitivity of the results to cloud retrievals during clear-sky scenes.

**Figure 1.**
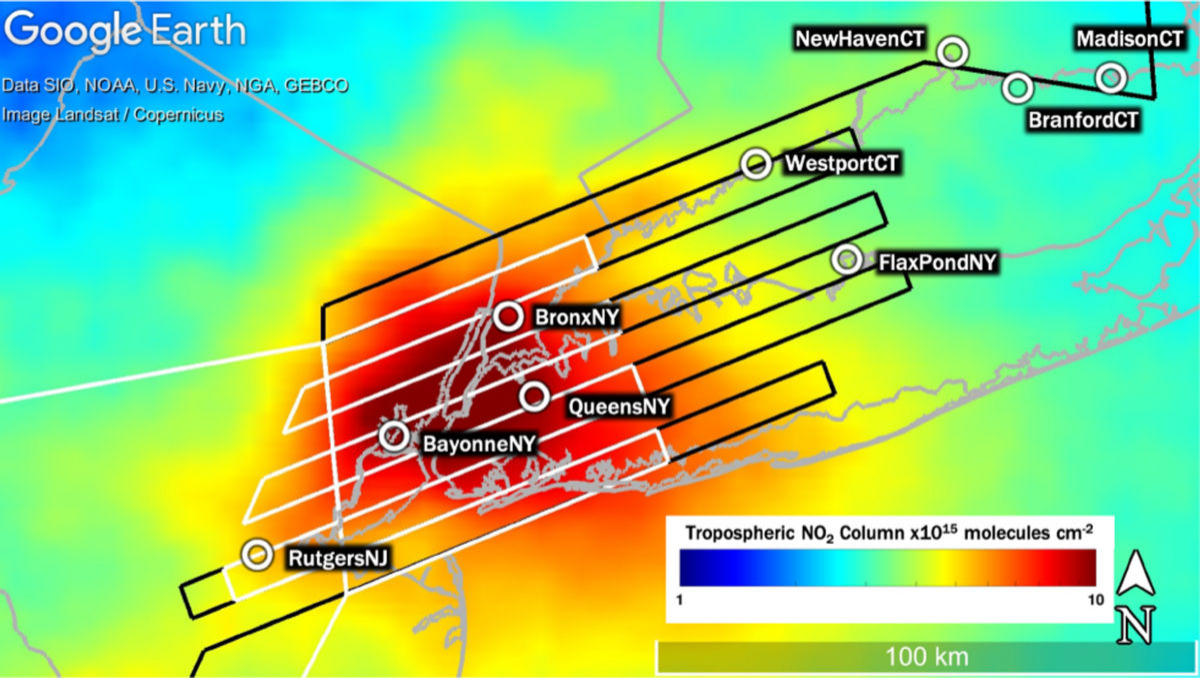
Map showing the annual average TROPOMI tropospheric NO_2_ columns between April 2018 and March 2019. Overlaid circles show the locations of the nine Pandora spectrometers considered in this analysis. Table 4 shows when each of these instruments operated. The black and white lines represent the two types of flight plans flown by the airborne spectrometers (large in black and small in white). This map was created in ©Google Earth Pro.

Figure 1 shows the annual average of NO_2_ TrVCs observed over the LISTOS region from April 2018 to March 2019, depicting peak NO_2_ in the domain of over 10×10^15^ molecules cm^−2^ over much of New York City. The largest value is over the southern tip of Manhattan Island at a magnitude of 12×10^15^ molecules cm^−2^. The spatial distribution and dynamic range of NO_2_ varies widely day to day over this region due to variable meteorology, emissions, and the lifetime of NO_2_, as shown through examples in this analysis.

### 2.3 Airborne spectrometers

Two airborne UV–VIS mapping spectrometers are used in this study: Geostationary Trace gas and Aerosol Sensor Optimization (GeoTASO) and GEO-CAPE Airborne Simulator (GCAS). GeoTASO and GCAS are very similar instruments but differ in characteristics such as their size, weight, wavelength range, and sensitivity. Specific details about these two instruments can be found in Leitch et al. (2014), Kowalewski and Janz (2014), Nowlan et al. (2016), and Nowlan et al. (2018), with a brief summary in Table 2. The two instruments have very similar performance with respect to the NO_2_ retrieval. Due to varying aircraft availability during LISTOS, these instruments were flown either interchangeably or together during 16 flight days between 18 June 2018 and 19 October 2018. Only flights from 25 June to 6 September (13 flight days) are considered in this analysis due to availability of the high-resolution model data used to provide the a priori NO_2_ profile shapes in the full vertical column retrieval (Table 1). GeoTASO was flown on the NASA LaRC HU-25 Falcon during the three June flight days, and GCAS was flown on the NASA LaRC B200 from July through October. The HU-25 Falcon is a faster aircraft (average ground speed at altitude was 215ms^−1^) capable of mapping approximately a 50% larger area per flight than the B200 (average ground speed at altitude was 123ms^−1^). This capability enabled us to also conduct measurements for the second Ozone Water-Land Environmental Transition Study domain (OWLETS2: https://www-air.larc.nasa.gov/missions/owlets/index.html: last access: 7 January 2020) during June flights over Baltimore, Maryland, in the early morning and late afternoon hours (outside the S5P overpass window). The NASA LaRC B200 has two nadir-viewing remote sensing portals, allowing installation of a second instrument along with GCAS. The second instrument from July through September was the High Altitude Lidar Observatory (HALO: Nehrir et al., 2018) providing colocated measurements of nadir profiles of aerosols and methane. This analysis uses HALO aerosol optical thickness (AOT) retrievals at 532 nm to discuss aerosol conditions qualitatively. GeoTASO was the second instrument for flights in October, allowing for direct comparison of GCAS and GeoTASO retrievals; however, these flights did not coincide with any clear-sky TROPOMI overpasses.

Figure 1 shows the two basic raster patterns that were flown by the NASA aircraft to create gapless maps of the high-spatial-resolution spectra from which NO_2_ TrVCs are retrieved. Both airborne instruments have a swath width of approximately 7 km at the nominal flight altitude of 9 km (aircraft indicated altitude of 28000 ft); thus, flight lines are spaced slightly over 6 km apart to ensure overlap between adjacent swaths. Table 3 includes a summary of all flights considered in this study along with cloud conditions, number of coincidences with Pandora and TROPOMI (assuming coincidence criteria discussed in Sect. 2.5 and throughout this paper), and raster type. All flight days included two flights lasting approximately 4–5 h each (morning and afternoon). The small raster (white lines in Fig. 1) could be accomplished two times in one flight (four times per day), repeatedly measuring the same area to observe the temporal variation throughout the day. The large raster (black lines in Fig. 1) could only be flown once per flight (twice per day) and was meant to capture a more regional view of the spatial distribution of NO_2_ on days with expected air pollution over Long Island Sound and the surrounding communities.

The NO_2_ retrieval algorithm is identical for GCAS and GeoTASO. The retrieval process is summarized here with additional detail in Judd et al. (2019). NO_2_ differential slant columns are retrieved at an approximate spatial resolution of 250m×250m in the spectral fitting window of 425–460 nm relative to in-flight-measured reference spectra using the open-source DOAS computing software, QDOAS (http://uv-vis.aeronomie.be/software/QDOAS/; last access: 18 April 2020). Reference spectra were collected over areas with low and homogeneous NO_2_ absorption over a 4–5 min time period using nadir observations for each of the 30 across-track positions. Three separate references were collected during the LISTOS campaign: 30 June for all GeoTASO flights, 2 July for the GCAS flights for this day only (due to unique instrument conditions), and 5 August for the rest of the GCAS flights as the instrument conditions were stable for the rest of the flight period. All reference spectra were colocated with total column NO_2_ measurements from Pandora spectrometers: 5.6×10^15^ molecules cm^−2^ at MadisonCT on 30 June, 5.7×10^15^ molecules cm^−2^ at MadisonCT on 2 July, and 6.2×10^15^ molecules cm^−2^ at WestportCT on 5 August, with values estimated to be over 50% stratospheric according to our TROPOMI bias-corrected stratospheric column estimation (see below).

Fitted trace gas absorption cross sections in the slant column spectral fit include NO_2_ (Vandaele et al., 1998), O_4_ (Thalman and Volkamer, 2013), water vapor (Rothman et al., 2009), CHOCHO (Volkamer et al., 2005), Ring spectrum (Chance and Kurucz, 2010), and a fifth-order polynomial. Average ±standard deviation spectral fitting uncertainties for the NO_2_ slant columns during cloud-free scenes at cruising altitude for GeoTASO are 1.6× 10^15^ ±0.3×10^15^ molecules cm^−2^ and for GCAS are 0.8× 10^15^ ±0.1×10^15^ molecules cm^−2^. The differences in uncertainty between spectral fits are likely due to a minor amount of undersampling of the GeoTASO slit function, which has a slightly flattened top hat shape compared to the more purely Gaussian shape exhibited by GCAS.

Air mass factors (AMFs) are calculated using the Smithsonian Astrophysical Observatory AMF tool (Nowlan et al., 2016, 2018), which packages the VLIDORT radiative transfer model (Spurr, 2006) for calculating scattering weights based on user inputs of viewing and solar geometries, a priori assumptions about surface reflectivity with bidirectional reflectance distribution function (BRDF) kernels, and meteorological and trace gas vertical profiles. AMFs are then calculated following the methodology of Palmer et al. (2001) as the integrated product of scattering weights and shape factor (e.g., Nowlan et al., 2016; Lamsal et al., 2017; Judd et al., 2019).

Table 1 compares a priori assumptions used for TROPOMI and airborne AMF calculations. For both retrievals, the spatial resolutions of the a priori assumptions are coarser than those of the observations, but a priori assumptions for airborne observations are at a finer resolution than those for TROPOMI. Airborne a priori NO_2_ vertical profile shapes are obtained for the troposphere from hourly output from a parallel developmental simulation of the North American Model–Community Multiscale Air Quality (NAMCMAQ) model from the National Air Quality Forecasting Capability (NAQFC; Stajner et al., 2011) and stratospheric NO_2_ climatology developed using PRATMO (PRather ATmospheric MOdel) (Prather, 1992; McLinden et al., 2000; Nowlan et al., 2016). The stratospheric column is bias corrected daily using TROPOMI NO_2_ stratospheric vertical columns by calculating the average offset between the two datasets over the LISTOS domain for each day (ranging from 5×10^13^ to 6×10^14^ molecules cm^−2^). This analysis only focuses on the below-aircraft portion of the NO_2_ columns from the aircraft, which is henceforth referred to as tropospheric vertical columns or TrVCs.

Surface reflectance over land is represented in the AMF tool input files with the isometric, geometric, and volumetric BRDF kernels given by the MODIS MCD43A1 product at 500 m resolution at 470 nm averaged over the time period of the LISTOS campaign (Lucht et al., 2000; Schaaf and Wang, 2015). Input over water includes only the isometric BRDF kernel, limited to a minimum of 3% Lambertian reflectivity (similar to Nowlan et al., 2016), as well as an added Cox–Munk kernel (derived through references from Cox and Munk, 1954; Nakajima and Tanaka, 1983; Gordon and Wang, 1992; Spurr 2014; and wind speed from the lowest layer of the NAMCMAQ model and viewing and solar geometry). The brighter areas where the isometric BRDF kernel exceeds 3% are mostly over lakes, rivers, and coastlines rather than open water. Water surfaces are flagged using the Terra MODIS Land-Water Mask MOD44W product.

A temperature correction is applied within the air mass factor calculation (e.g., Bucsela et al., 2013) as the slant column retrievals only use an NO_2_ absorption cross section at one temperature (294 K). The temperature correction factor is the same factor used in the TROPOMI NO_2_ product (van Geffen et al., 2019).

Clouds or aerosols are not accounted for in the AMF calculation in this analysis, though cloudy scenes are excluded from the analysis using a defined count rate threshold measured by the airborne spectrometer detector and visual verification from GOES 16 imagery (https://www.star.nesdis.noaa.gov/smcd/spb/aq/AerosolWatch/; last access: 18 April 2020).

Differential slant columns are converted to below-aircraft vertical columns (assumed as the tropospheric vertical column, TrVC) by subtracting the estimated stratospheric slant column (PRATMO climatology bias corrected daily with TROPOMI multiplied by the stratospheric AMF), adding the estimated reference slant column amount (from Pandora), and dividing by the tropospheric air mass factor, similar to Eq. (1) in Judd et al. (2019) or Eq. (4). in Nowlan et al. (2018).

Previous work quantified uncertainty in airborne TrVCs from GCAS and GeoTASO by applying error propagation through the calculation of the vertical column based on uncertainties in the slant column fit, reference spectrum, and AMF calculation (Nowlan et al., 2016, 2018; Judd et al., 2019). Relative uncertainties are largest for relatively clean sites (up to and over 100% in individual cases); however, they decrease as pollution increases. Lorente et al. (2017) found that different methodologies applied to the same datasets can lead to structural uncertainty of 31%–42%, which is mostly due to sensitivity to selection of a priori vertical profile shapes in the AMF calculation. In this work, airborne TrVCs are evaluated by comparing to Pandora NO_2_ columns (Sect. 3) as Pandora NO_2_ columns have relatively low uncertainties and their AMFs are not dependent on a priori profile shapes as described in the following section.

### 2.4 Pandora spectrometers

The Pandora instrument is a ground-based UV–VIS spectrometer that provides high-quality spectrally resolved direct-sun/lunar or sky scan radiance measurements. The Pandora radiance measurements combine trace gas spectral fitting routines and, in the case of sky scan measurements, radiative transfer models to provide column densities of trace gas species similar to TROPOMI and airborne spectrometers. Pandora measurements obtained throughout the LISTOS study were limited to direct-sun mode, during which the instrument tracks the sun to observe the direct solar irradiance. Direct-sun columns are particularly beneficial for validation/evaluation due to their low uncertainties in the AMF (Herman et al., 2009). All data are processed as part of the Pandonia Global Network (PGN; https://www.pandonia-global-network.org/, last access: 6 November 2020), and only data with a quality flag of 0 or 10 (high quality) are used. Accuracy and precision of the total NO_2_ column measurements from Pandora are reported as 2.69×10^15^ molecules cm^−2^ for an AMF of 1 and 1.35× 10^14^ molecules cm^−2^, respectively (Herman et al., 2009; LuftBlick, 2016). All Pandora data are converted from total vertical columns to TrVCs by subtracting either the airborne-estimated or TROPOMI-retrieved stratospheric columns for comparison purposes.

Nine Pandora spectrometers were deployed and operated in the LISTOS domain in support of the LISTOS air quality study and as long-term measurements in support of EPA’s Photochemical Assessment Monitoring Station Enhanced Monitoring (PAMS-EM) program (https://www3.epa.gov/ttnamti1/files/ambient/pams/PAMSEMPGuidance.pdf; last access: 24 March 2020). Here, we use available Pandora data from these nine instruments between June 2018 and March 2019. There is one additional long-term Pandora located in NYC (CCNY campus, Instrument PI: M. Tzortziou) that is not part of the PAMS-EM program and thus is not included in the quantitative analysis presented here. However, this instrument is used briefly to describe a case study in Sect. 4.

The names, locations, and monthly days of operation of the nine Pandora spectrometer sites used in this analysis are shown in Table 4. Figure 1 also shows the spatial distribution of these sites, which includes one site to the west of NYC (RutgersNJ), three instruments within the New York City metro area (BayonneNJ, BronxNY, and QueensNY), and five along the shoreline of Long Island Sound to the east-northeast of the city. Pandora sites were chosen to capture upwind, in-city, and downwind emissions from NYC, particularly NO_2_ transport down Long Island Sound from the city to help investigate the complex ozone pollution near this land–water interface. All instruments operated during the summer 2018 LISTOS campaign (defined as through September 2018), though four sites operated beyond LISTOS and are used in Sect. 5.2 for evaluation through 19 March 2019.

### 2.5 Methods

All linear regression statistics in this work are calculated using a reduced major axis (RMA) including the coefficient of determination (*r*^2^). This regression was chosen over ordinary least squares (OLS) to recognize the potential for uncertainty in both evaluated and reference measurements. Percent and mean differences are also calculated and analyzed and are calculated by the following convention:

(1) columndifference=evaluatedmeasurement-referencemeasurement,

(2) $$\;\text{percent}\;(\text{\%})\;\text{difference}\;=\frac{\;\text{column}\;\text{difference}\;}{\;\text{reference}\;\text{measurement}\;}\times 100.$$

In Sects. 3 and 5, the reference measurements are the Pandora TrVCs and the evaluated measurements are the airborne and TROPOMI TrVCs, respectively. In Sect. 4, the reference measurements are the aircraft TrVCs and the evaluated measurements are TROPOMI NO_2_ columns.

For all comparisons, coincidence criteria are chosen based on spatial, temporal, and physical components of the evaluated and reference measurements. In the following analysis, we use the following coincidence criteria (unless otherwise noted).

For Pandora and airborne coincidences, the recommended coincidence criteria are from Judd et al. (2019), which are the median airborne TrVCs within a 750m radius of the Pandora site and the temporally closest Pandora measurement (within ±5 min of the aircraft overpass).

For airborne comparisons to TROPOMI, each TROPOMI pixel must be at least 75% mapped by cloud-free airborne pixels within ±30 min of the S5P overpass.

- For Pandora comparisons to TROPOMI, the coincidence is identified by the TROPOMI pixel in which the Pandora spectrometer is located (according to the TROPOMI pixel corners) and the median Pandora TrVC is calculated within ±30 min of the S5P overpass.
- All TROPOMI data have cloud radiative fractions (CRFs) less than 50%. An additional new criterion is invoked to exclude points for which the difference between surface pressure and cloud pressure in the retrieval (as an indication of cloud height) exceeds 50 hPa. Justification of this criterion is discussed primarily in Sects. 4.1 and S3, and the influence of the criterion is considered throughout the paper.
- Sensitivities to coincidence criteria are detailed in Tables S1–S3 and briefly discussed in each section and within the Supplement to this paper.
- In addition to the standard TROPOMI v1.2 NO_2_ TrVC product we consider the effect of using a higher-spatial-resolution a priori NO_2_ vertical profile shape in the TROPOMI retrieval. This is done by recalculating TROPOMI tropospheric AMF using the tropospheric averaging kernel to replace the TM5-MP a priori profile with the 12 km NAMCMAQ data used in the airborne spectrometer AMF calculations following the guidance provided in Sect. 8.8 of Eskes et al. (2019).

## 3 Evaluating airborne TrVC with Pandora data

This work begins by comparing airborne and Pandora TrVC to evaluate the uncertainty of the airborne TrVCs and establish the spatial representativeness of the Pandora observations. This evaluation provides a consistent basis for using the high-spatial-resolution airborne data and high-temporal-resolution Pandora data to independently assess TROPOMI TrVCs.

**Figure 2.**
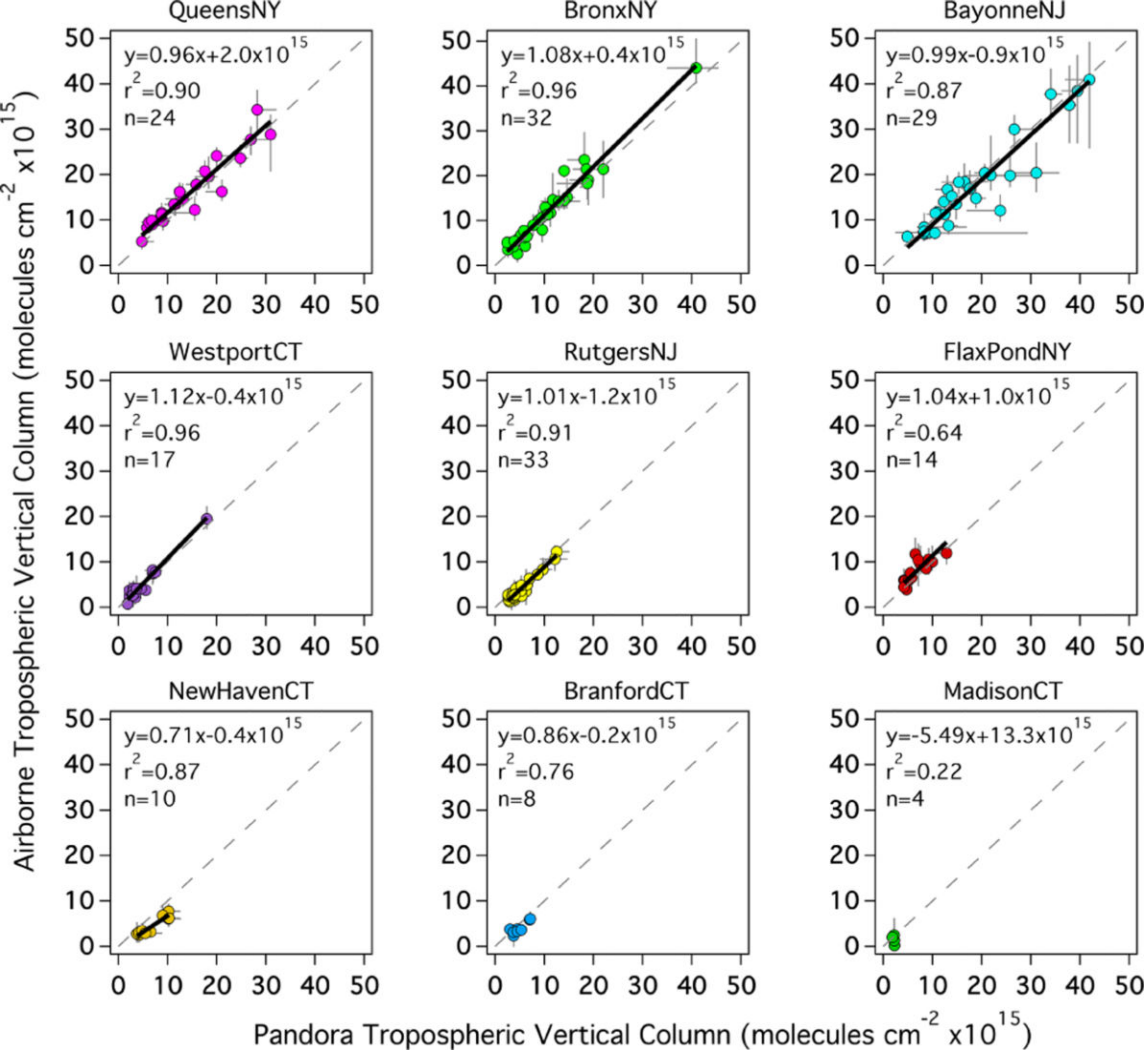
Scatter plots of the temporally closest Pandora TrVC to the aircraft overpass (±min/max observation within a ±5 min window from the aircraft overpass) vs. median airborne TrVC within a 750 m radius of Pandora (±10th–90th percentile) with labeled statistics. The 1 : 1 line is indicated with the grey dashed line. The solid black lines indicate the RMA linear regression for sites with *r*^2^ greater than 0.5.

During LISTOS, overflights of Pandora sites with the airborne spectrometers occurred during all 13 flight days spanning 25 June–6 September 2018, between 12:00 and 22:00 UTC (08:00–18:00 EDT). Site-by-site scatter plots of all coincident measurements and linear regression statistics are shown in Fig. 2. At most sites the Pandora and airborne tropospheric NO_2_ columns are highly correlated with slopes of approximately 1. Bars extending from each coincidence illustrate the spatial and temporal variability at the time of the measurements; the horizontal bars show the maximum and minimum Pandora observations within ±5 min of the aircraft overpass, and the vertical bars show the 10th–90th percentiles of the airborne pixels within a 750 m radius of the Pandora site (usually ∼ 25–30 pixels). High temporal and spatial variations are mostly observed at polluted locations (e.g., QueensNY, BronxNY, and BayonneNJ). NewHavenCT has the lowest slope (0.71) of all sites yet a high correlation (*r*^2^ =0.87) which suggests a possible systematic site bias. Such a bias could be due to the inability of the MODIS BRDF product to resolve the spatial gradient of surface reflectance near this site, as this site is adjacent to both a bright urban area in New Haven and also the darker surface of the nearby river. Excluding MadisonCT, which has a poor linear regression due to the few (4) coincidences and small data range, the *y* intercepts of the linear regressions range from −1.2×10^15^ to 2.0×10^15^ molecules cm^−2^. The most likely cause for the range in *y* intercepts between sites would be uncertainty in the estimated column for the reference spectrum in the Pandora retrieval, which uses the minimum Langley extrapolation (MLE) approach and has an estimated accuracy of 2.69×10^15^ molecules cm^−2^ for an AMF of 1 (Herman et al., 2009). The observed intercepts are all smaller than this estimated uncertainty.

**Figure 3.**
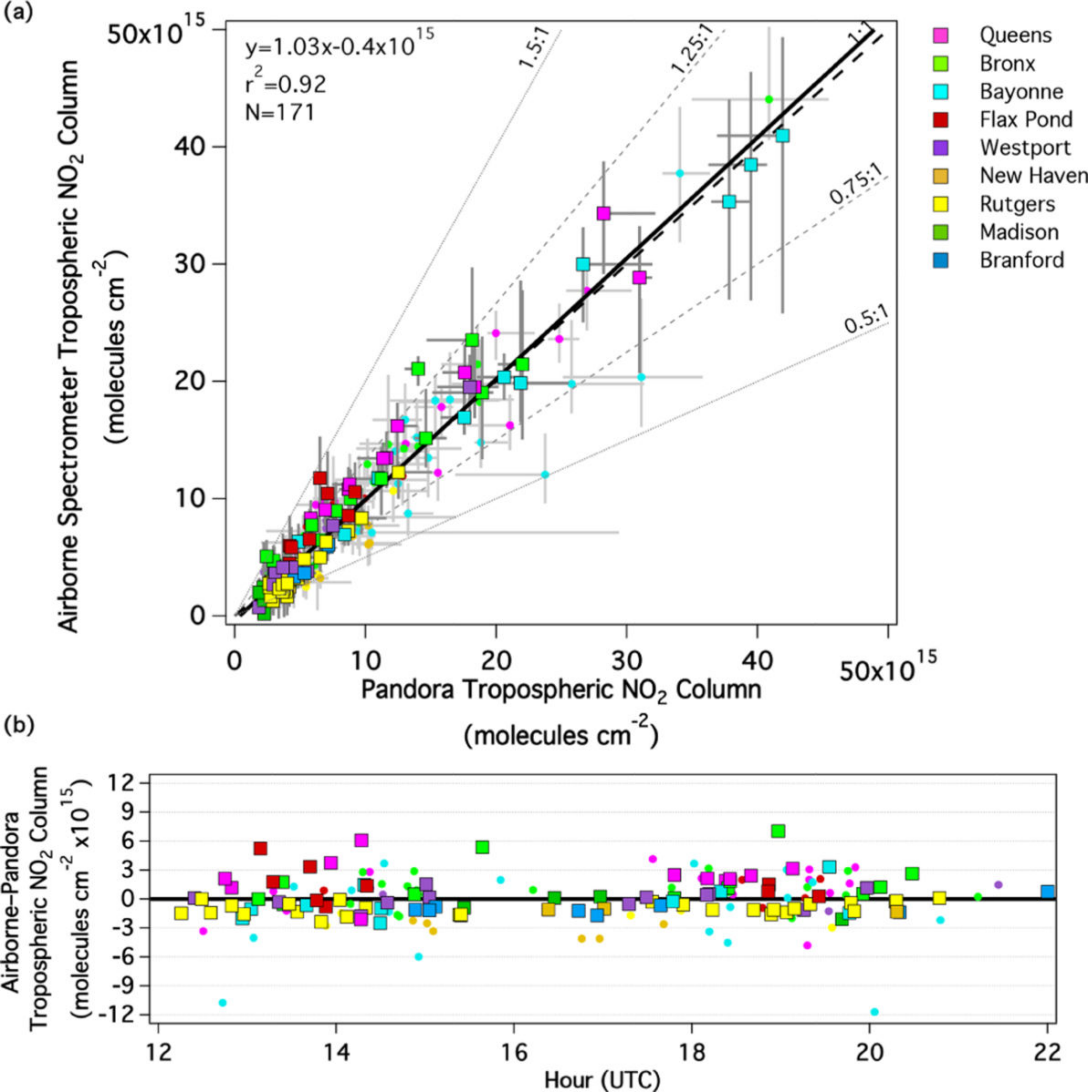
**(a)** Scatter plot showing the temporally closest Pandora TrVC to the aircraft overpass (±min/max observation within a ±5 min window from the aircraft overpass) vs. the median airborne TrVC (±10th–90th percentile) within a 750 m radius of the Pandora site. The thick solid black line represents the RMA linear regression. Each point is colored by Pandora location, where the outlined squares are points where Pandora TrVCs do not vary more than 30% within a ±15 min window from the aircraft overpass, whereas the circles indicate times where Pandora TrVCs do vary more than 30%. ***(b)*** The difference between airborne and Pandora tropospheric NO_2_ columns vs. time of day in hours (UTC) colored similarly to ***(a).***

Figure 3 shows the aggregated comparison of airborne and Pandora TrVC coincidences from all sites during LISTOS (*n*=171). Figure 3a shows the scatter plot and linear regression statistics. Each point is colored by the Pandora location, consistent with Fig. 2. Together, these data are highly correlated (*r*^2^ =0.92) with a slope of 1.03 and small offset of −0.4×10^15^ molecules cm^−2^. Figure 3a also includes whiskers showing the spatial and temporal variability associated with each coincident observation similar to Fig. 2. Two different symbols are used as an objective indicator of temporal variability as quantified by Pandora observations; the outlined squares in Fig. 3a are coincidences where the Pandora TrVCs vary less than 30% within ±15 min from the aircraft overpass (*n*=97), and the nonoutlined circles indicate those exceeding 30% (*n*=74). (The temporal window for this assessment is larger than the ±5 min shown in the max/min horizontal whiskers to include more data points to assess temporal variability.) Most of the temporally homogeneous points tightly span the 1 : 1 relationship, with 95% falling within ±25 % or having a difference less than 2.69×10^15^ molecules cm^−2^. More of the temporally variable points expand further from the 1 : 1 line though still mostly fall within ±50% or have a difference less than 2.69×10^15^ molecules cm^−2^ (98%). Considering only the temporally homogeneous measurements results in a very similar RMA fit (slope and offset) and a distinctly improved *r*^2^ (0.96 vs. 0.92) but a loss of 43% of the number of data points (compare Table S1 row H to row B). This demonstrates the potential benefit of the high temporal resolution of Pandora observations for evaluating the impact of heterogeneity in NO_2_ comparisons.

Previous work has suggested that the azimuth direction of the Pandora observation (due to its sunward-viewing observations) can impact comparisons to airborne spectrometers in heterogeneously NO_2_ polluted regions (Nowlan et al., 2018; Judd et al., 2019). We assessed this directionality sensitivity by also examining subsets of the airborne data within sectors surrounding Pandora’s azimuth pointing direction (±22.5 and ±45^◦^ sectors were considered). The sector constraint slightly degrades the linear regression statistics, with an increase in slope of 4%–5%, decrease in *y* intercept of 2–3×10^14^ molecules cm^−2^, and no change in correlation (Table S1, compare rows D and E to row B). Considering directionality of Pandora can still be important in assessing individual cases but is not broadly implemented in this analysis due to the relative insensitivity found here and the limited feasibility of doing it in comparisons with the more spatially coarse measurements from satellites (including TROPOMI).

While most of the temporally homogeneous points are within ±25% of each other, there are a small number of coincidences where the airborne spectrometer retrievals are more than 25% larger than Pandora. There were no clouds during these coincidences. The two Bronx coincidences that fall near the 1.25 : 1 line both occurred on 2 July 2018 during the morning and afternoon flights. The viewing direction of Pandora toward the southeast in the morning along with elevated NO_2_ to the west of the site can partially explain the differences in the morning flight (as indicated by the large vertical whiskers for the green box near an airborne TrVC of 23×10^15^ molecules cm^−2^), though in the afternoon NO_2_ is more homogeneous spatially near this location. Aerosols are elevated over the site on this day (HALO-measured AOT at 532 nm is ∼ 0.3), which could lead to a high bias in airborne TrVCs due to an underestimation in the AMF. However other coincidences during LISTOS also occurred with AOT of 0.3 or larger, and there is no apparent correlation between AOT and the airborne/Pandora differences (Fig. S1). Other coincidences on 2 July (*n* = 7) do not show a systematic aircraft high bias. The other temporally homogeneous high outlier occurred at Flax Pond on 29 August 2019 just after 13:00 UTC, with no explanation related to the viewing direction of Pandora and no elevated aerosols (AOT∼0.16). This coincidence has the lowest calculated airborne tropospheric AMF (0.53), which may be too low due to the a priori profile being strongly weighted toward the surface than it is in reality. The NAMCMAQ TrVC at this time is 1.7×10^16^ molecules cm^−2^, where 84% of that NO_2_ is below 300ma.g.l., suggesting too much near-surface NO_2_ in this a priori profile. Less NO_2_ near the surface in this a priori profile would increase the tropospheric AMF calculation at this site, and a tropospheric AMF of 0.83 would bring this point into agreement with Pandora. The most likely reason for all these differences is incorrect vertical distribution and magnitude of NO_2_ by the NAMCMAQ model and its influence on the tropospheric AMF (which would need to increase 27%–64% to bring these cases into agreement with Pandora).

Figure 3b shows the difference between the airborne and Pandora observations as a function of time of day. Overall, there does not appear to be a dependence on time of day, which gives confidence that the airborne retrievals are correctly representing the effects of viewing and solar geometrical input, varying NO_2_ a priori profiles through the day due to dynamic mixing and the growth of the boundary layer, and varying surface reflectivity based on the MODIS BRDF data in the radiative transfer model. Most (81%) of these differences are within ±2.69×10^15^ molecules cm^−2^ – the quoted accuracy of Pandora NO_2_ retrievals in Herman et al. (2009). These results are encouraging for future validation studies of retrievals from data collected aboard geostationary platforms (e.g., TEMPO; Zoogman et al., 2017) with these types of airborne measurements. Considering only those coincidences during the overpass window of S5P (Table S1, compare row B to row I) slightly improves the correlation (*r*^2^ increases from 0.92 to 0.94) but degrades the slope and intercept (slope increases from 1.03 to 1.13 with a compensating decrease in the *y* intercept from −0.4 to −1.1×10^15^ molecules cm^−2^). However, the median percent difference from Pandora is only 2% during this time period.

**Figure 4.**
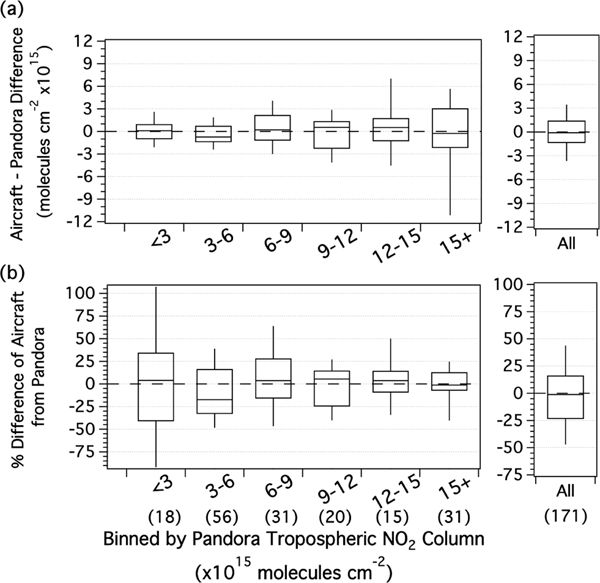
Box plots (95, 75, 50, 25, 5) showing the airborne column ***(a)*** column difference and ***(b)*** percent difference from Pandora binned at the labeled thresholds (×10^15^) as well as all data points (right). The number of points in each bin is indicated by the numbers in parentheses above the **x** axis label.

Figure 4 assesses the uncertainty of the airborne data and its potential sensitivity to pollution level. For the least polluted columns (below 3×10^15^ molecules cm^−2^), the interquartile range of the column difference is within ±1×10^15^, with a median of 0.1×10^15^. For the more polluted columns, the interquartile range of the percent difference is mostly within 25%, with a median difference within 0.6×10^15^ molecules cm^−2^. These conclusions are not dependent on choice of reference (i.e., the results are similar if examined as a function of binned airborne TrVC). For all data, the median percent difference is −1% with an interquartile range of −23% to 16%.

Considering all results between Pandora and the airborne spectrometers, uncertainty in the airborne spectrometer TrVC NO_2_ is generally within ±25% with no obvious bias overall. This uncertainty is lower than estimated using error propagation in previous literature, suggesting the errors in a priori datasets are smaller than was estimated in each study (Nowlan et al., 2016, 2018; Judd et al., 2019).

## 4 Evaluating TROPOMI TrVC with airborne data

**Figure 5.**
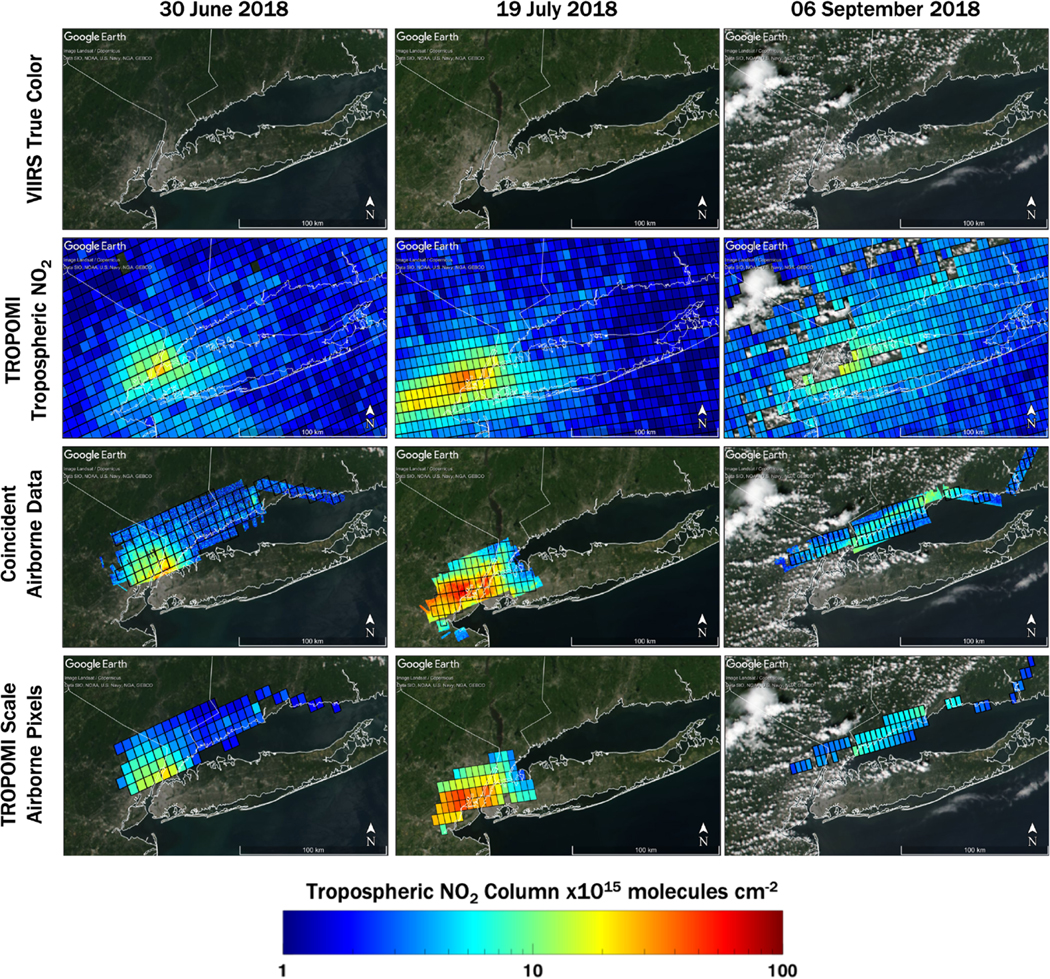
Maps demonstrating how airborne data are matched to TROPOMI for 3 out of 15 example overpasses: (top) VIIRS true color imagery (source: https://worldview.earthdata.nasa.gov/: last access: 18 April 2020), (second row) overlaid TROPOMI TrVCs where CRFs<50%, (third row) overlaid airborne data collected within ±30 min of the TROPOMI overpass with outlined TROPOMI pixels with CRFs<50% and area mapped by aircraft >75%, and (bottom) airborne NO_2_ column data scaled to the TROPOMI pixel. All maps were created in © Google Earth Pro.

Airborne spectrometer data provide a spatially representative dataset in which to compare to TROPOMI with added information about subpixel variability. During the LISTOS campaign, flight plans were designed with the intent to be airborne at the time of the S5P overpass. Figure 5 illustrates how the airborne data are matched to TROPOMI coincidences during three separate orbits – 30 June, 19 July, and 6 September. The maps on the top row are true color imagery from the Visible Infrared Imaging Radiometer Suite (VIIRS) sensor which overpasses approximately 5 min before S5P (data source: https://worldview.earthdata.nasa.gov/, last access: 6 November 2020), showing that the first 2 d were clear of clouds but cumulus clouds were present during the 6 September overpass. The second row shows the overlaid TROPOMI TrVCs. NO_2_ data are colored on a log10 scale spanning 1–100×10^15^ molecules cm^−2^. These three cases illustrate how the day-to-day changes in spatial patterns and the dynamic range of NO_2_ can be dramatically different from the annual average shown in Fig. 1 (note difference in color bar ranges between Figs. 5 and 1).

To compare the two datasets, coincident data following appropriate spatial, temporal, and other physical characteristics are extracted as discussed in Sect. 2.5. The third row in Fig. 5 shows the airborne data that match the temporal coincidence criteria for these three orbits (±30 min from the S5P overpass). The black outlines show TROPOMI pixels that are at least 75% mapped by the airborne spectrometers during this temporal window. Visually, the spatial patterns in TrVC observed by TROPOMI and the airborne instrument are consistent with each other. Finally, the subpixel airborne data within each TROPOMI pixel are gridded to a 250 m matrix to account for overlapping data from adjacent swaths, and then the area-weighted averages of the airborne TrVCs are computed to create values that are spatially and temporally consistent with the TROPOMI TrVC observations (bottom row in Fig. 5; gridding methodology from Kim et al., 2016).

From 25 June to 6 September 2018, the airborne spectrometers collected data that coincided with over 1300 TROPOMI pixels within ±30 min of the S5P overpass. However, when considering only pixels 75% mapped by the airborne spectrometer and with CRF less than 50%, the number of coincidences decreases to 621. Additionally, through this analysis, we found that several notable outliers (coincidences with large apparent differences between the two measurements) corresponded with cloud retrieval effects in cloud-free scenes. Therefore, one additional coincidence criterion is applied to include only scenes with differences between the cloud pressure and surface pressures (Δ_CS_) less than 50hPa (the reported uncertainty of the cloud pressure retrieval in van Geffen et al., 2019). This criterion eliminates any TROPOMI pixels with assumed clouds and results in a reduction in the number of data points to 388. The impact of this criterion is discussed in Sect. 4.1, with an illustrative case study in Sect. S3 in the Supplement, though points exceeding this coincidence criteria are still shown in scatter plots throughout this paper as blue crosses. (Statistics without this criterion are shown within Tables 5 and 7 and in the Supplement).

**Figure 6.**
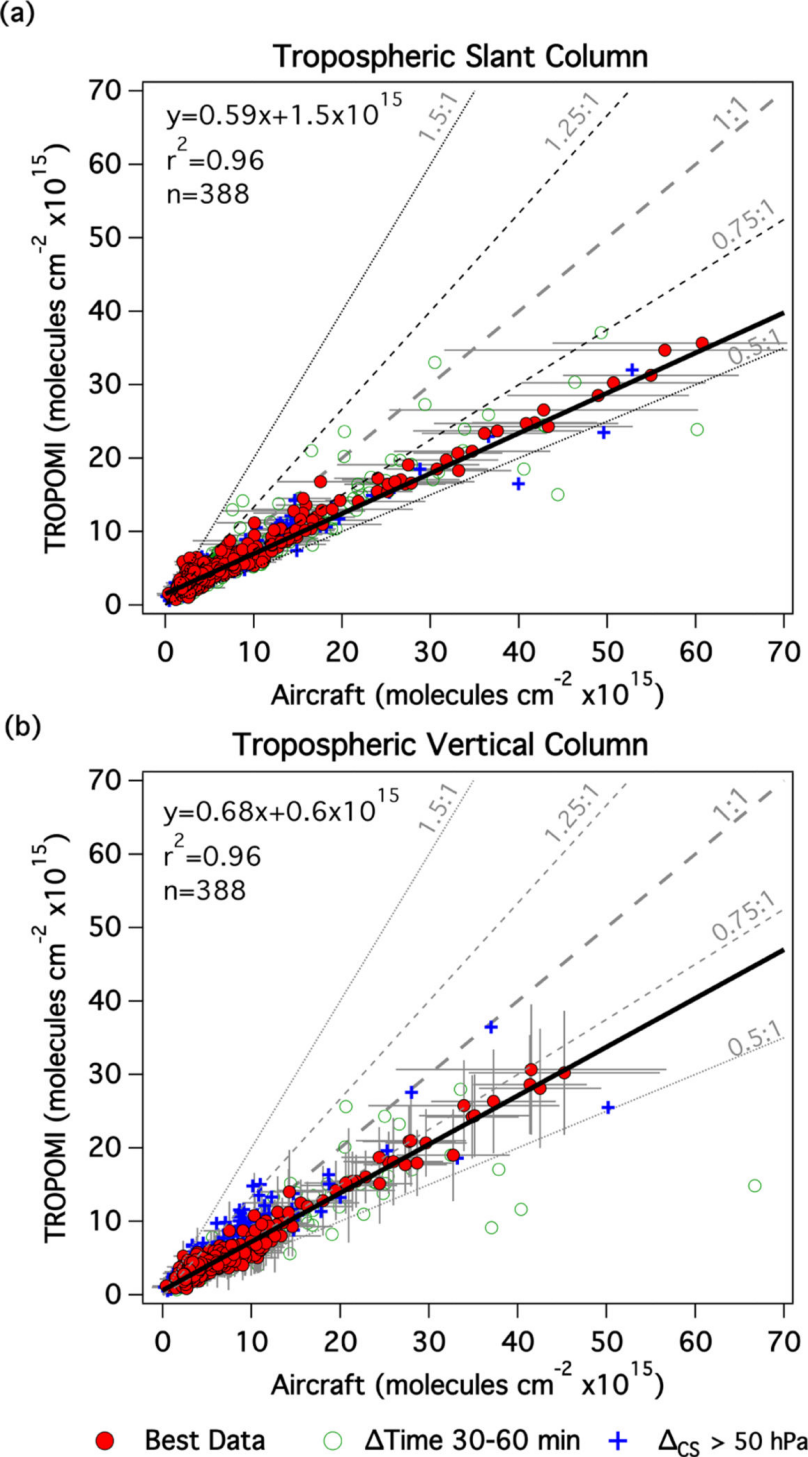
Scatter plots of airborne data gridded and scaled up to the TROPOMI pixel footprint vs. TROPOMI NO_2_ tropospheric ***(a)*** slant column and ***(b)*** vertical column that are at least 75% mapped with a CRF<50 % within ±30 min of the TROPOMI overpass in red circles (open green circles show points when the time window is expanded to ±60 min, and blue crosses symbolize points where Δ_CS_ >50 hPa). The horizontal bars indicate the subpixel heterogeneity measured by the aircraft quantified as the standard deviation of aircraft slant columns over that pixel, and vertical bars in panel ***(b)*** show the reported precision of the TROPOMI TrVC (the precision of the tropospheric slant columns in panel ***(a)*** is not large enough to be visible in this figure, but the average is 5×10^14^ molecules cm^−2^ with a standard deviation of 7×10^13^ molecules cm^−2^).

Figure 6 shows scatter plot and linear regression statistics of all slant and vertical column coincidences between TROPOMI and the airborne data. The red circles in these plots represent the data that meet the strictest coincidence criteria discussed in the previous paragraph. For these points, the slant columns are very highly correlated (*r*^2^ = 0.96). TROPOMI slant columns are consistently smaller than the airborne spectrometer slant columns (slope=0.59), though airborne slant columns are expected to be larger in comparison to satellite observations because the airborne spectrometers are more sensitive to altitudes nearer to the surface (where much of the NO_2_ resides) due to the lower observational altitude of the aircraft. However, as shown by the high correlation, TROPOMI and the aircraft are sampling nearly the same atmosphere, at least in the lowest parts of the atmosphere that make up the majority of the TrVC. Converting from slant to vertical column increases (improves) the regression slope by 15% while preserving the very high correlation (*r*^2^ = 0.96).

While the remaining low bias reflected by the slope below the 1 : 1 line will be discussed in subsequent subsections, we first begin with some discussion about potential reasoning for the small amount of scatter that exists between the TROPOMI and airborne measurements. These causes include (1) a spatial component (i.e., we allow TROPOMI-scale airborne pixels to be missing data in up to 25% of the area of the TROPOMI pixel), (2) a temporal component as we allow up to 30 min difference between the time of the measurements, and (3) differing a priori assumptions made within each retrieval.

Considering the spatial component of scatter, the horizontal bars in Fig. 6 show the standard deviation of the subpixel airborne TrVCs within each TROPOMI pixel. Generally, the variation in subpixel NO_2_ increases as the NO_2_ TrVC increases, illustrating how scatter in the comparisons could increase if only small subsets of the pixel are mapped. Sensitivity to the mapped percentage is annotated in Table S2 (rows B–D and M–O) and shows little impact when relaxing the percent-mapped criterion to 50% (though it is impacted negatively when the Δ_CS_ criterion is applied; Table S2: rows M–O) and a more significant decrease when relaxing to 25%. At least with the airborne samples in this case the linear statistics are driven by the most polluted pixels that are 100% mapped by the airborne spectrometers, explaining the limited sensitivity in the RMA fit to the percentage of the TROPOMI pixel mapped in this study.

Addressing the temporal component, if the temporal window is decreased to ±15 min from ±30 min, the number of mapped TROPOMI pixels by the aircraft decreases by 65% while the quality of linear statistics is moderately improved (Table S2, compare row B to row E). However, there is a larger adverse impact to the RMA fit and *r*^2^ when the time window is extended to extract airborne data within ±60 min of the S5P overpass. Coincidences occurring between 30 and 60 min from the S5P overpass are shown as open circles in Fig. 6. For example, the small subset of very polluted airborne TrVCs that are much larger than what is retrieved by TROPOMI occurred during a time with high temporal variability on 2 July 2018. The airborne spectrometer observed a distinct very polluted plume over NYC and over the 48 min period between the airborne and TROPOMI observations, and the Pandora spectrometer located at CCNY observed a 50% decrease in NO_2_ total vertical column, leading to a large difference between the airborne and TROPOMI TrVCs when the temporal window is extended to ±60 min (Maria Tzortziou, personal communication, 8 August 2020).

These outliers are caused by real spatiotemporal variability rather than issues in either of the retrievals and demonstrate the care needed for matching airborne data collected over time to the nearly instantaneous observations from S5P TROPOMI. These large differences are also apparent in the slant column comparisons, and future studies should consider slant column comparison between aircraft and TROPOMI as a guide for identifying potential spatial and temporal mismatches.

With respect to differing retrieval assumptions, we consider two factors in the following subsections: treatment of clouds and NO_2_ vertical profile shape.

### 4.1 Cloud retrieval effects

In previous literature, a coincidence criterion based on CRF from TROPOMI has been the common consideration for data comparisons, though studies vary slightly in their chosen CRF threshold (ranging from 30%–50% in Griffin et al., 2019; Ialongo et al., 2020; and Zhao et al., 2020). We investigate the effect on the statistics of varying CRF threshold, alone, but find that retrieved cloud height is also an important factor and here consider the two effects together.

In the TROPOMI retrieval, surface reflectivity is estimated using the 0.5°×0.5° climatology from 5 years of OMI observations (Kleipool et al., 2008; van Geffen et al., 2019). When the surface albedo climatology used for TROPOMI has a low bias, which can occur over bright city centers, the algorithm increases the overall brightness of the scene by assuming a nonzero cloud fraction. In cloud-free urban scenes, this approach generally results in a nonzero CRF with a nominal cloud pressure equal to the surface pressure. Figure S2a illustrates this behavior on a cloud-free day (19 July 2018).

This CRF-adjustment approach over bright surfaces generally appears to work well; however, we identified a potential issue when the retrieval also places retrieved clouds above the surface rather than at the surface in cloud-free scenes. The two most obvious illustrations of this effect are evident as the two blue crosses farthest above the regression line with airborne TrVCs greater than 25×10^15^ molecules cm^−2^ in Fig. 6. Section S3 presents a case study demonstrating that the effect is correctable for these two points. We note that, in the presence of significant scattering aerosols, CRF may also be larger than zero and the cloud pressure level may mimic the height of the aerosol layer. During aircraft coincidences with TROPOMI, the average AOT at 532 nm measured by HALO was 0.22 with a standard deviation of 0.15. In the case of these outliers, elevated aerosol loading has been ruled out (AOT at 532 nm was 0.04). Clouds and their effect on the estimated vertical sensitivity are an important component within the NO_2_ retrieval, as clouds are assumed to shield the view of the atmosphere below the cloud level in some fractions of the pixel. However, in cloud-free scenes, cloud pressures significantly less than the surface pressure with elevated CRF can lead to an underestimation in the AMF, and therefore an overestimation in TROPOMI TrVC, as the shielding that is assumed through the retrieval is not occurring in reality. Because the airborne screening criteria ensure that only cloud-free observations are included in our analysis, our comparisons are biased toward cloud-free scenes, and therefore high CRFs are associated generally with bright surfaces instead of clouds.

To avoid these impacts, we explored an additional coincidence criterion based on cloud parameters in the TROPOMI product file. We consider an allowable difference between retrieved cloud pressure and surface pressure (henceforth Δ_CS_) of less than 50 hPa (which is the reported uncertainty in cloud pressure retrieval from van Geffen et al., 2019). Figure 6 shows points that exceed this criterion as blue cross symbols, and the linear regression statistics with and without this criterion applied are summarized in Table 5. Applying this criterion removes approximately 30% of coincidences including the largest outliers but also many points that are not outliers. Of the 233 data points that have Δ_CS_ greater than 50 hPa, 58% (*n* = 136) of them have aircraft-measured cloud fractions of less than 2%, and 69% of these cloud-free coincidences (*n* = 94) have reported CRFs greater than 10%, illustrating that the cloud retrieval regularly yields an effective cloud height above the surface even during cloud-free scenes. Further filtering data by only removing data with CRFs > 10% results in very little change in the overall statistics. Table 5 shows that the largest impact of the Δ_CS_ criterion is an improvement in the correlation (*r*^2^ of 0.96 vs. 0.90) but a slope further from 1 (0.68 vs. 0.71) and a more negative median percent difference (−19% vs. −11%), showing that there is excellent correlation between the two measurements but an apparent low bias in the TROPOMI retrieval that the cloud pressure errors partially offset. This impact is also confined to the TrVC comparisons and not apparent in the slant column comparisons, which demonstrates the impact is through assumptions made in the AMF calculation.

Eskes and Eichmann (2019) mention occurrences of negative effective cloud fractions in the FRESCO cloud product that could also result in positive cloud fraction in the NO_2_ window in v1.2 of the TROPOMI TrVC product, which causes a noisy NO_2_ retrieval. The occurrence of negative FRESCO cloud fractions with positive CRFs did occur during many of these coincidences (63% of the 621 pixels). However, this fraction is much lower for Δ_CS_ flagged pixels (18%), and they were not associated with the largest outliers in this analysis. Applying a criterion to remove negative cloud fractions instead of Δ_CS_ flagged pixels results in similar results to only filtering for CRFs<50% and no Δ_CS_ criterion (slope=0.72, offset=0.7×10^15^ molecules cm^−2^, *r*^2^ = 0.91, and *n* = 233). Therefore, this impact is not the cause for the described patterns in the previous paragraph.

In the vertical columns, coincidences identified by the Δ_CS_ criterion typically lie above the best-fit line, consistent with the hypothesis of effective cloud shielding in the AMF calculation during cloud-free scenes. There is one obvious coincidence exceeding the Δ_CS_ threshold that opposes this general pattern by falling below the best-fit line (blue cross with airborne TrVC around 50×10^15^ molecules cm^−2^). This apparent disparity appears to be caused by large temporal variation between the times of the airborne and satellite measurements. The airborne measurement preceded TROPOMI by 23 min, and in a subsequent airborne measurement over the same area 70 min later, the airborne NO_2_ TrVC had decreased to approximately 30×10^15^ molecules cm^−2^, which is much nearer to the TROPOMI-measured value of 25×10^15^ molecules cm^−2^. This is another example where a temporal mismatch resulted in an outlier in the slant column comparisons in Fig. 6a demonstrating the use of slant column comparisons to assist in identifying spatial and temporal mismatches.

Finally, we summarize the sensitivity to different CRF thresholds. Without the Δ_CS_ criterion applied (Table S2; rows F–I), allowing larger CRF values generally decreases *r*^2^ while increasing the slope slightly and dramatically increasing the number of coincidences. The highest correlations, up to 0.96, are maintained with CRF<20%. When the Δ_CS_ threshold is applied, the RMA fit is largely insensitive to changes in CRF up to 50% (Table S2: rows J–M), maintaining the high quality of the linear regression while including progressively more data points with increasing CRF thresholds. Because CRF can often exceed 20% over urban areas even in cloud-free conditions due to effects of the coarse a priori surface reflectivity used in the retrieval, the Δ_CS_ criterion appears useful for retaining valid cloud-free coincidences over bright urban scenes. Overall, the best fit is attained either by restricting CRF to less than 20% and not using the Δ_CS_ criterion or by using the Δ_CS_ criterion, which allows inclusion of CRF values up to 50% and provides 35% more coincidences. Future research could explore using alternative cloud measurements (e.g., from VIIRS) to identify cloud-free scenes and the use of clear-sky AMFs.

### 4.2 NO_2_ vertical profile shape

The a priori vertical profiles in the TROPOMI NO_2_ retrieval are from the TM5-MP model with a spatial resolution of 1° ×1° interpolated to the center of the TROPOMI pixels (van Geffen et al., 2019). In a heterogeneously polluted region such as NYC, NO_2_ profiles vary at much smaller spatial scales. For spatial reference, the airborne spectrometer flights for each LISTOS raster (Fig. 1) cover an area of approximately 1° ×1° or smaller, and airborne TrVCs span up to 2 orders of magnitude in this domain. Here, TROPOMI tropospheric AMFs are recalculated with the 12 km NAMCMAQ analysis used in the airborne TrVC retrieval to demonstrate the impact of spatial resolution of a priori profiles. These TROPOMI TrVCs columns are hereafter labeled as TROPOMI-NAMCMAQ. The original TROPOMI v1.2 product is referred to as TROPOMI standard.

**Figure 7.**
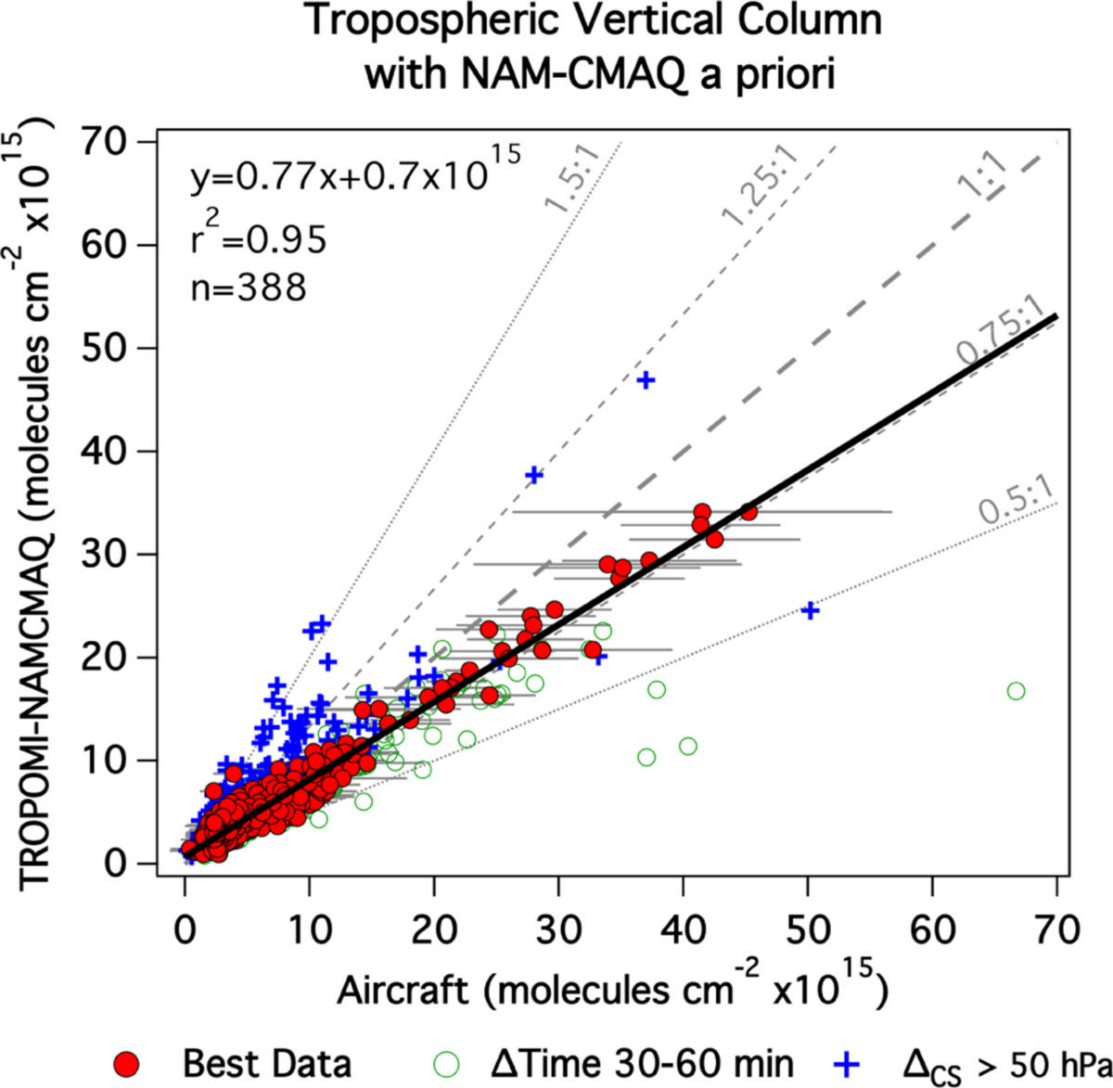
Scatter plots of airborne data gridded and scaled up to the TROPOMI pixel footprint vs. TROPOMI-NAMCMAQ NO_2_ TrVCs that are at least 75% mapped with a CRF<50% within ±30 min of the TROPOMI overpass in red circles (open green circles show points when the time window is expanded to ±60 min, and blue crosses symbolize points where Δ_CS_ >50 hPa). The horizontal bars indicate the subpixel heterogeneity measured by the aircraft quantified as the standard deviation of aircraft vertical columns over that TROPOMI pixel.

Figure 7 has the same format as Fig. 6 but instead compares TROPOMI-NAMCMAQ to airborne TrVCs. (Note that both datasets are now using the same a priori profiles.) In general, applying the NAMCMAQ profile to the TROPOMI AMF calculation brings the airborne and TROPOMI data into closer agreement; with the Δ_CS_ criterion applied, the slope increases 13% from 0.68 to 0.77, the median percent difference improves from −19% to −7%, and a high *r*^2^ is maintained (changing from 0.96 to 0.95).

Incorporating a higher-resolution a priori profile appears to result in an increase in the sensitivity to the Δ_CS_ criterion, with more of the blue cross points visible in Fig. 7 than in Fig. 6, which can likely be attributed to increased sensitivity to the lower altitude levels in the AMF calculation. In the higher-resolution NAMCMAQ analysis, the lower levels are more polluted and thus more sensitive to cloud shielding.

**Figure 8.**
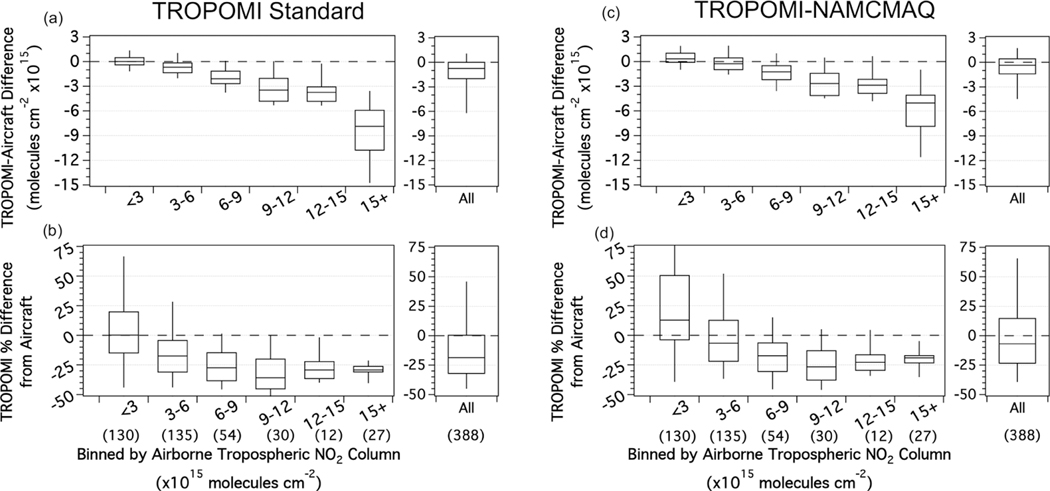
Box plots (95, 75, 50, 25, 5) showing the TROPOMI TrVC ***(a)*** column difference and ***(b)*** percent difference from airborne TrVCs binned at the labeled thresholds (×10^15^) as well as for the total dataset (right), along with the equivalent box plots for TROPOMI-NAMCMAQ in panels ***(c)*** and ***(d)***. The number of points in each bin are indicated by the numbers in parentheses above the **x** axis label.

The biases of the TROPOMI standard and TROPOMI-NAMCMAQ TrVCs with respect to the airborne data are further examined as a function of pollution level in Fig. 8. The majority of points (68%) are less than 6×10^15^ molecules cm^−2^, so the overall distributions are dominated by the behavior in the lowest bins in Fig. 8. In these lowest two bins, the median percent difference is −10% and +3%, respectively, for TROPOMI standard and TROPOMI-NAMCMAQ TrVCs. Column differences unsurprisingly increase with pollution level and are small in these two lowest bins, with the interquartile range within 1×10^15^ molecules cm^−2^ and inner 90% of points having differences within 2×10^15^ molecules cm^−2^. TROPOMI standard has a median absolute bias of zero in the lowest bin. Using the NAMCMAQ profile shifts the bias more positive in all bins, creating a small positive bias in the lowest bin but reducing the overall median bias from −1×10^15^ molecules cm^−2^ to 0.3×10^15^ molecules cm^−2^. For airborne TrVCs above 6×10^15^ molecules cm^−2^, the median percent difference is −29% for the TROPOMI standard but improves to −20% for TROPOMI-NAMCMAQ. Although a higher-resolution a priori profile improves the overall bias in the TROPOMI product, there is still a low bias for the most polluted TROPOMI TrVCs columns.

## 5 Evaluating TROPOMI TrVC with Pandora data

**Figure 9.**
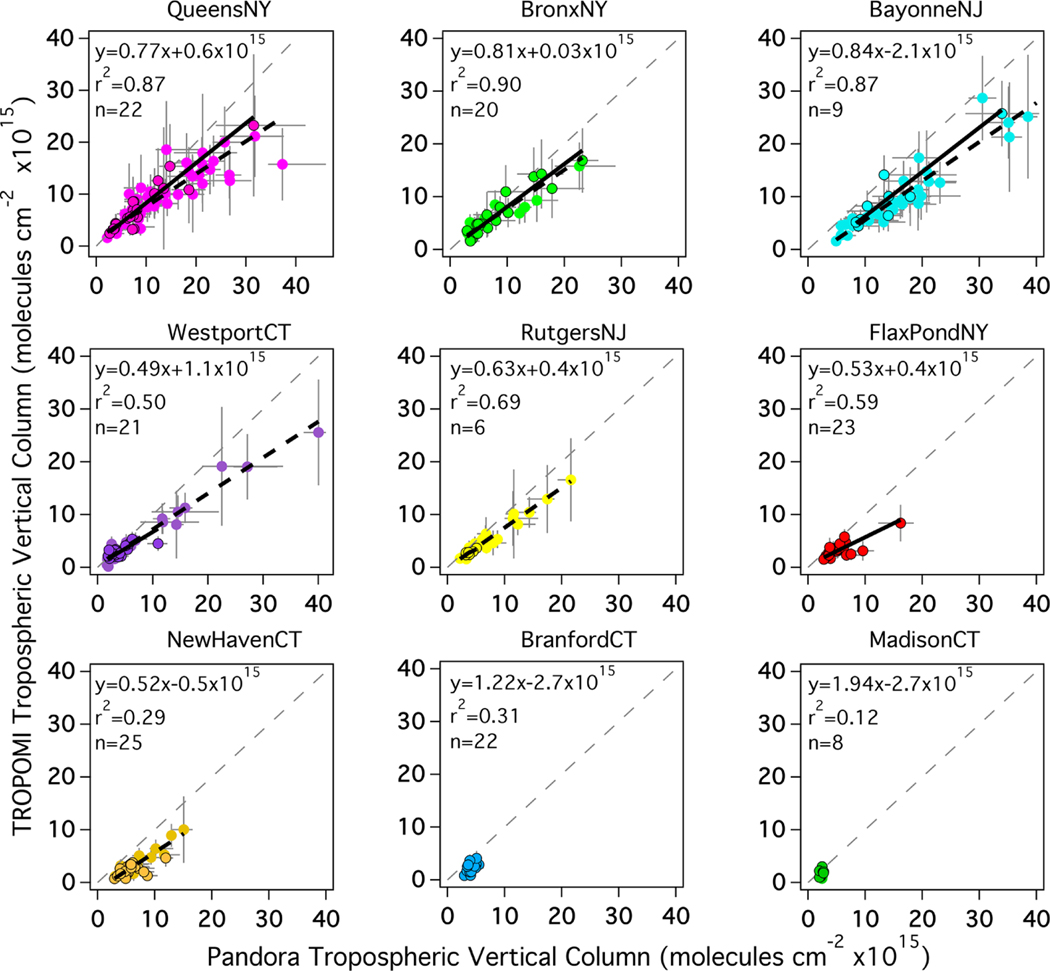
Scatter plots of the median Pandora TrVC within ±30 min of the S5P overpass vs. TROPOMI TrVC for all coincidences with CRF<50% and Δ_CS_ <50 hPa between 25 June 2018 and 19 March 2019 at each individual site. Coincidences during the LISTOS intensive period (through the end of September 2018) are outlined in black. Vertical bars indicate the reported precision of TROPOMI TrVCs, and the horizontal bars are the 10th–90th percentile of Pandora TrVCs within ±30 min of the S5P overpass. The 1 : 1 line is indicated with the grey dashed line. Statistics are summarized in Table 6, but the RMA regression lines are shown for datasets with *r*^2^ greater than 0.5 (solid black line is for the LISTOS timeframe and dashed black line is all data).

Pandora spectrometers operated in the LISTOS domain during and after the conclusion of the intensive LISTOS airborne measurements as part of the PAMS-EM program (see Table 4). Following coincidence criteria in line with those from Sect. 4 (TROPOMI CRF<50%, Δ_CS_ less than 50 hPa, and median Pandora TrVC within ±30 min), Fig. 9 shows all coincidences between Pandora and TROPOMI through 19 March 2019, with coincidences during the LISTOS intensive period (defined as any measurements prior to and including 30 September 2018) outlined in black. Site-by-site statistics are listed in Table 6 for both time periods. In this section we discuss consistency in TROPOMI evaluation results with airborne spectrometers using data from only the LISTOS time period and also from an extended temporal window at select sites that operated through winter 2019.

### 5.1 TROPOMI vs. Pandora during LISTOS

During the LISTOS time period, there were 156 coincidences between the nine Pandora spectrometers and TROPOMI, ranging from 8 to 25 coincidences by site (Table 6). With the exception of MadisonCT and BranfordCT (which lack in TrVC dynamic range), the slope of TROPOMI vs. Pandora is less than 1 (ranging from 0.49 to 0.84, similar to the results in Sect. 4) with moderate to high values of *r*^2^ (0.29–0.90). All median percent differences are negative and vary by site ranging from −9% to −52%.

**Figure 10.**
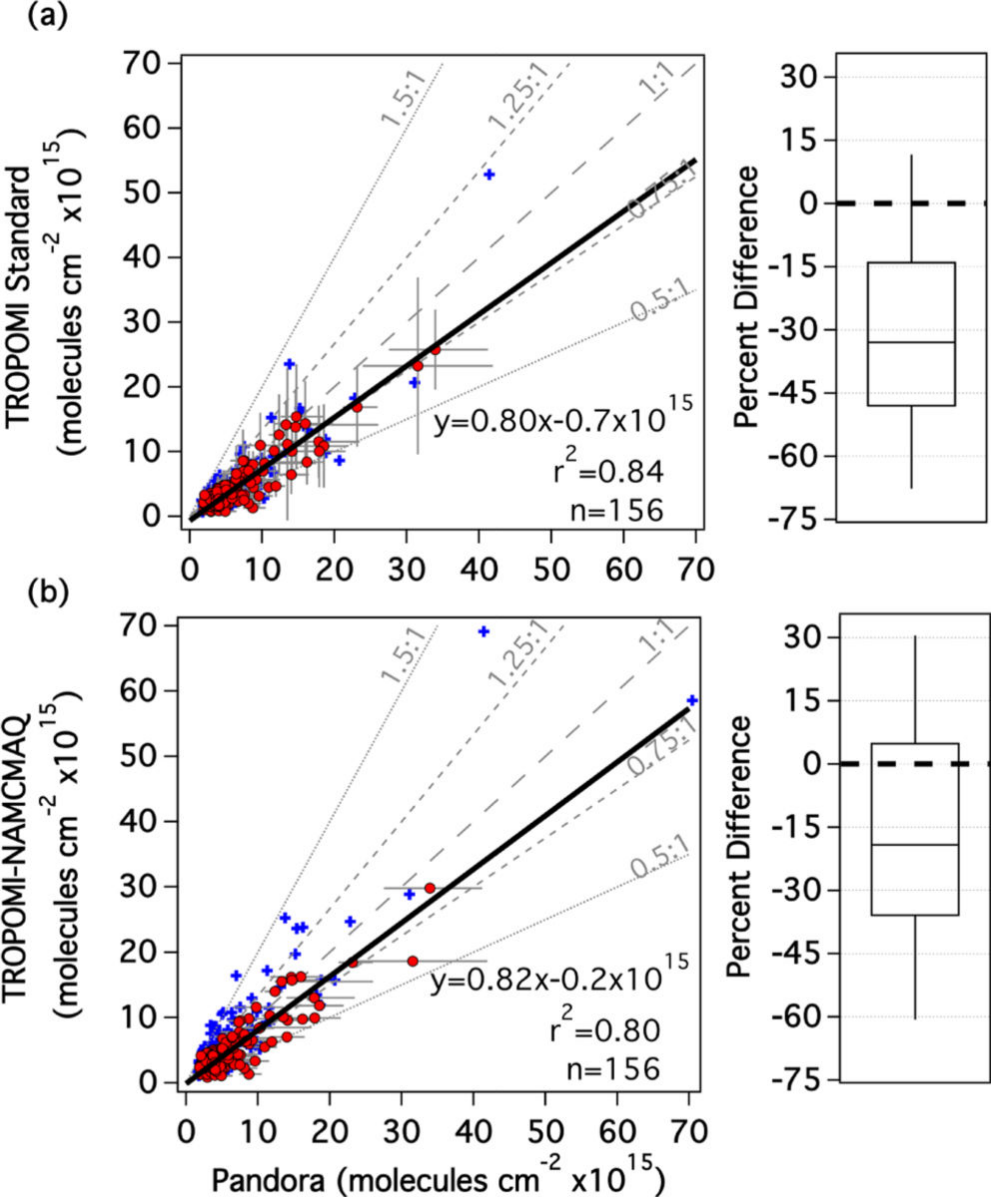
Scatter plot showing coincident ***(a)*** TROPOMI standard TrVCs and ***(b)*** TROPOMI-NAMCMAQ TrVCs with CRF<50% vs. median Pandora NO_2_ TrVC over a ±30 min temporal window during the LISTOS intensive period. Red points have a Δ_CS_ <50 hPa, whereas blue crosses have a Δ_CS_ >50 hPa. The horizontal bars represent the 10th–90th percentile of Pandora data within the ±30 min temporal window. The vertical bars in panel ***(a)*** represent the reported precision of TROPOMI standard. The thick solid black line represents the RMA linear regression applied to the red data points. The box plots (95, 75, 50, 25, 5) show the TROPOMI TrVC percent difference from Pandora for the red data points to the right of each scatter plot.

Figure 10a shows the aggregated TROPOMI standard and Pandora dataset during LISTOS; red circles/blue crosses are those that have a Δ_CS_ less than/greater than 50 hPa, respectively, similar to Fig. 6. The bars represent the reported precision of the TROPOMI standard product (vertical) and the 10th–90th percentile of Pandora data within the ±30 min window (horizontal). Temporal variation of TrVCs measured by Pandora increases proportionally to pollution level (*r*^2^ = 0.69). The aggregated dataset shows that TROPOMI TrVCs have a low bias in comparison to Pandora (slope=0.80 and offset of −0.7×10^15^ molecules cm^−2^) and high correlation (*r*^2^ =0.84). As a whole, TROPOMI has a median percent difference from Pandora of −33% with an interquartile range of −48% to −14%, consistent with comparisons of TROPOMI to airborne TrVCs for values above 6×10^15^ molecules cm^−2^. Comparing Figs. 10a to 6b, the slope is 18% higher (better) than in the comparisons to the TROPOMI standard product to airborne TrVCs, though at the expense of a lower *r*^2^ (0.96 vs. 0.84). Coincidences at QueensNY and BronxNY have the lowest median percent difference of all the sites, and the aggregate slope is sensitive to whether these two sites are included or not (0.80 and 0.72 with and without BronxNY and QueensNY, respectively). This result highlights the sensitivity of site selection and duration in the combined analysis and can likely be attributed to differences in spatial representativity between the TROPOMI and Pandora and perhaps sampling temporally over just the short period of the LISTOS study.

Spatial representativity of Pandora and subpixel variation in the TROPOMI area can also influence the results. TROPOMI pixels span an areal coverage of approximately 30–130 km^2^ depending on the position in the swath through S5P’s 16 d orbit cycle, while Pandora measurements represent a more localized environment. We found that the interquartile range of the TROPOMI bias relative to Pandora becomes slightly more negative as the pixel size gets larger (not shown). For pixels less than 40 km^2^, the interquartile range is −1% to −46% (*n* = 67), whereas for pixels larger than 80 km^2^, it is −14% to −59% (*n* = 18).

Unlike with airborne spectrometer data comparisons, sub-TROPOMI pixel cloud information is not readily available for these comparisons to Pandora. However, the impact of coincidence criteria based on clouds is assessed similarly to Sect. 4. Lowering of the CRF threshold preferentially excludes data from sites with brighter surface reflectivity and, typically, larger NO_2_ values. For example, QueensNY has a median CRF of 34% (minimum of 17%), whereas a more rural location like WestportCT has a median CRF of 8% (minimum of 0%). Without applying the Δ_CS_ criterion, we find the quality of the linear regression statistics to be quite sensitive to CRF threshold (Table S3, rows F–I). Using more restrictive CRF thresholds generally worsens the correlation, and the trends here are less consistent than found in the TROPOMI-airborne comparisons. This inconsistency is due to the relatively fewer number of Pandora coincidences having large values, e.g., above 10×10^15^ molecules cm^−2^, which makes the linear regression sensitive to screening criteria such as CRF that exclude any of the larger-valued data points. Though applying the Δ_CS_ criterion removes nearly half the coincidences for CRFs<50%, its application increases *r*^2^ values at all CRF thresholds (Table S3; rows J–M). Applying the Δ_CS_ criterion maintains high correlations while allowing retention of data from bright urban sites that would be preferentially left out by filtering by CRF for thresholds 30% and lower.

Figure 10b shows the comparison between TROPOMI-NAMCMAQ TrVCs and Pandora. Many more coincidences with Δ_CS_ greater than 50 hPa (blue crosses) are evident above the 1 : 1 line, again illustrating the increased sensitivity to this parameter when higher-resolution a priori profiles are used within the TROPOMI AMF calculation. Table 7 summarizes all the various cases. Considering all coincidences without invoking the Δ_CS_ criterion (i.e., including blue crosses and red circles), there is a large improvement in the regression statistics from TROPOMI standard to TROPOMI-NAMCMAQ, with the slope closer to 1 and a median percent difference of only −9% (relative to the −30% for TROPOMI standard). However, as illustrated by the blue points in Fig. 10b, it is clear that this improvement is partially driven by a high bias related to the impact of clouds. When points with Δ_CS_ greater than 50 hPa are excluded, the slope between TROPOMI-NAMCMAQ and Pandora improves by only 2.5% in comparison to TROPOMI standard, with a slight degradation of *r*^2^ from 0.84 to 0.80. However, there is a large improvement in the median percent difference, from −33% (interquartile range of −48% to −14%) for TROPOMI standard to −19% (interquartile range of −36% to 5%) for TROPOMI-NAMCMAQ.

Much of the correlation in Fig. 10 is driven by the 20 points above 10×10^15^ molecules cm^−2^; considering only points below 10×10^15^ molecules cm^−2^ lowers *r*^2^ to 0.42 and 0.39 for TROPOMI standard and TROPOMI-NAMCMAQ, respectively, though this results in the same median percent differences. The loss in correlation demonstrates the challenge of doing linear regressions on datasets with a lack of dynamic range well above 10×10^15^ molecules cm^−2^ in this analysis when spatiotemporal variability impacts can be at a similar magnitude. However, extending analysis through winter 2019 results in a larger sampled dynamic range as demonstrated in the next section.

### 5.2 TROPOMI vs. Pandora through 19 March 2019

**Figure 11.**
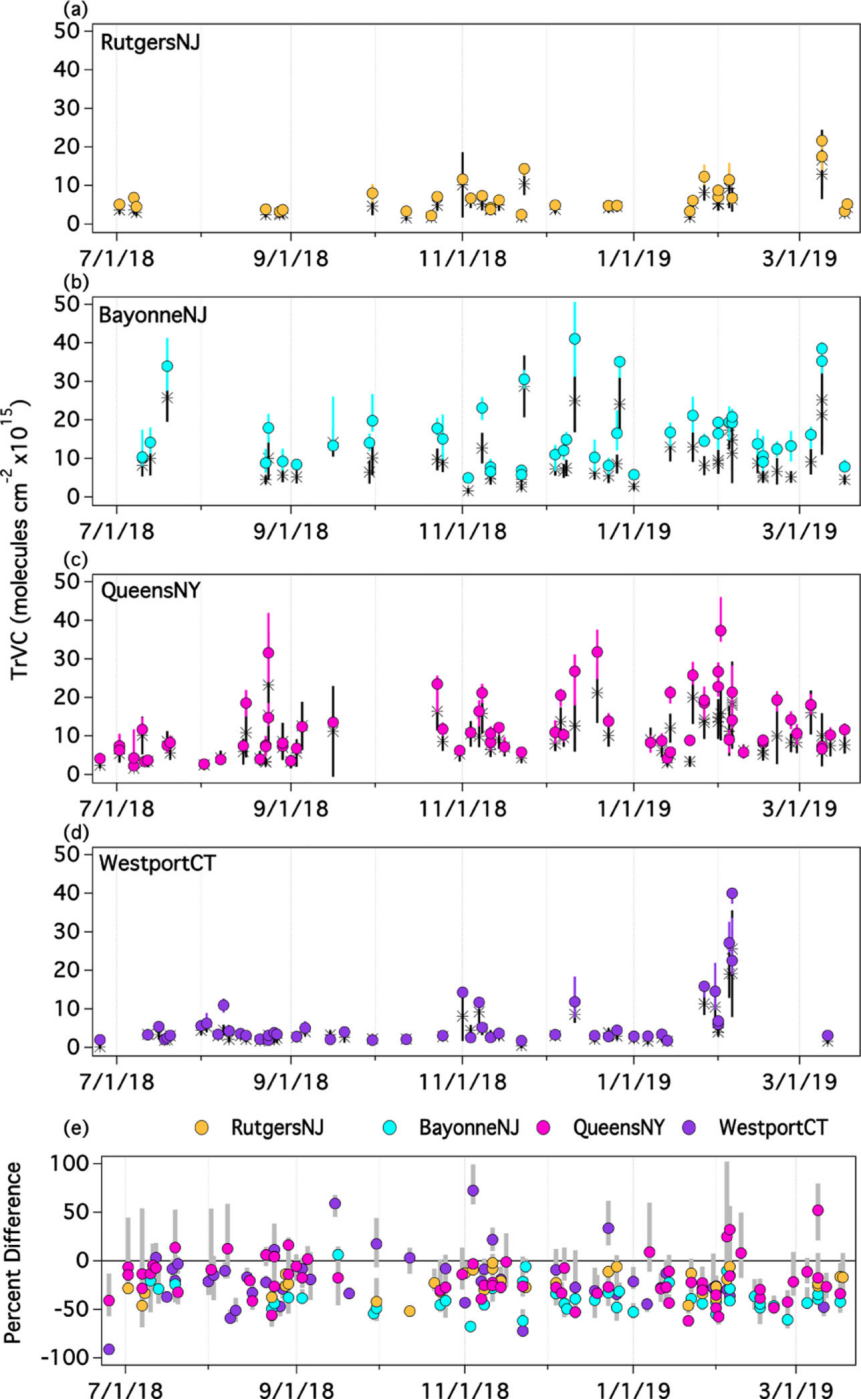
Time series of Pandora and TROPOMI standard TrVCs from 25 June 2018 through 19 March 2019. Circles represent the Pandora data ±10th–90th percentile in the ±30 min window and the stars indicated the TROPOMI TrVC ± the reported precision at ***(a)*** RutgersNJ, ***(b)*** BayonneNJ, ***(c)*** QueensNY, and ***(d)*** WestportCT. The percent difference of the TROPOMI standard TrVC from Pandora colored by site is shown in panel ***(e)***, and the grey bars indicate the 10th–90th percentile of the column difference of TROPOMI TrVC from the subtemporal Pandora data.

The deployment of many of the Pandora instruments in this region as part of the PAMS-EM program presents the opportunity for evaluation beyond the period of the LISTOS intensive campaign. TROPOMI level 2 NO_2_ processing switched to version 1.3 after 19 March 2019; thus, this analysis goes only through this date to avoid possible influences associated with the version change. To ensure consistent spatial representativity through the period, analysis is limited to the four sites that continued operation through 19 March 2019 (Table 4; RutgersNJ, BayonneNJ, QueensNY, and WestportCT). The focus of this extended analysis is to see whether conclusions made from the LISTOS time period are still valid through the fall and winter months as photochemistry and meteorological changes lead to potential shifts in spatial and temporal variation and dynamic range at these sites. These four sites represent two in-city sites and sites upwind and downwind from NYC, though the upwind/downwind side of the city is dependent on wind direction from day to day. Figure 11 shows time series of Pandora and TROPOMI standard TrVCs from 25 June 2018 through 19 March 2019 at each of the sites. Colored circles represent the Pandora measurements during the S5P overpass, the black stars show the TROPOMI TrVC, and the whiskers indicate variability or uncertainty (see figure caption). Note that some days have two overpasses. In general, temporal patterns are similar in both TROPOMI and Pandora measurements, demonstrating each instrument’s ability to observe synoptic and seasonal variability in TrVCs.

At RutgersNJ and WestportCT, Pandora and TROPOMI TrVCs rarely exceed 10×10^15^ molecules cm^−2^ during the year. More polluted coincidences occurred periodically during November–March as expected given the longer photochemical lifetime of NO_2_ during winter. In early January, when both Pandora and TROPOMI values were low, the spatial distribution of NO_2_ in the LISTOS domain from TROPOMI showed that the NYC plume was advected over the Atlantic Ocean on most of these days and was not intercepted by either site. At WestportCT, there was an extended period of elevated columns near the end of January and beginning of February. The larger TrVC values during that period coincide with days when the NYC plume extends toward Long Island Sound and Connecticut, likely driven by synoptic flow from the southwest quadrant. (This is the flow orientation that is often linked with poor ozone air quality along the shorelines of Long Island Sound during the summertime, e.g., the late August 2018 timeframe which was active with respect to ozone (airnow.gov: last accessed 11 March 2019) but did not result in an NO_2_ enhancement over WestportCT, likely due to the shorter NO_2_ lifetime in summer.) Alternatively, at RutgersNJ on the 9 March, the Pandora site was encompassed by an NO_2_ plume extending from the center of NYC during two consecutive TROPOMI overpasses leading to its maximum TrVC values during the time period assessed. Unlike the other two sites, BayonneNJ and QueensNY have large dynamic ranges in NO_2_ TrVCs in all seasons due to their proximity to strong sources within the NYC metropolitan area. Extending comparisons through the winter allows for more frequently measuring large values to extend the dynamic range of the coincident measurements.

Figure 11e shows the percent difference in TROPOMI TrVCs from Pandora with the bars showing the temporal variability of these percent differences during the ±30 min temporal window from the S5P overpass (10th–90th percentile). Despite some changes seasonally in the magnitude of NO_2_ at each of the sites, the percent difference in TROPOMI from Pandora does not have an apparent significant trend over this time period. The majority of points fall within 0% to −50%. The points with percent differences closest to zero, including points with positive percent differences, are associated with small values at WestportCT. Many of the coincidences have very large ranges in percent difference due to the temporal variability of Pandora TrVCs within the ±30 min time period that are likely associated with subpixel heterogeneity, again illustrating the challenge of quantifying biases with Pandora in urban environments.

**Figure 12.**
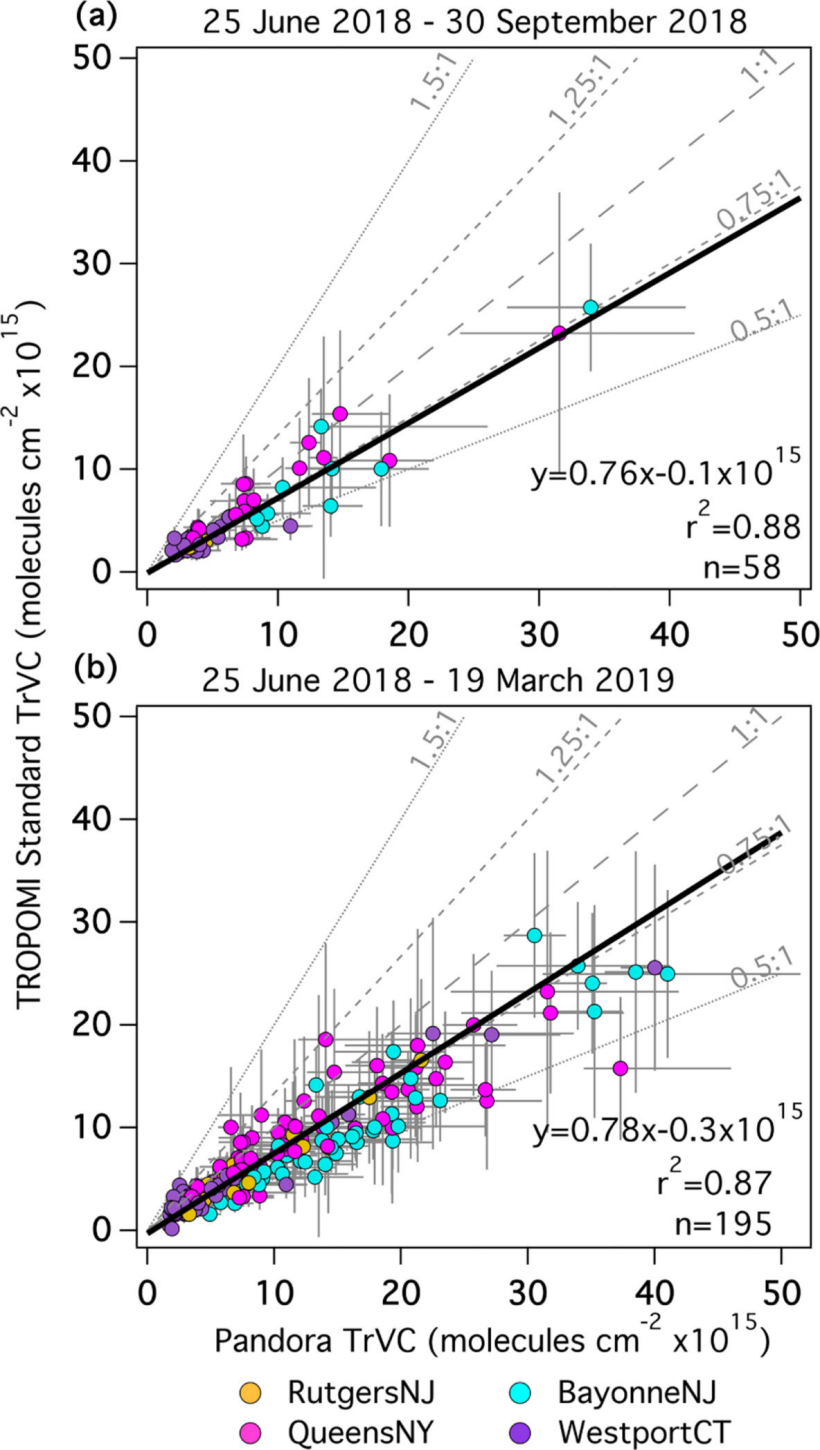
TROPOMI standard vs. Pandora TrVCs colored by site during ***(a)*** the LISTOS intensive period for the four locations with extended measurements in time (RutgersNJ, BayonneNJ, QueensNY, WestportCT) followed by ***(b)*** coincidences extending from 25 June 2018 to 19 March 2019 at the same four sites. The horizontal bars represent the 10th–90th percentile of Pandora data within the ±30 min temporal window. The vertical bars represent the reported precision of TROPOMI. Each point is colored by Pandora location.

Figure 12 shows a scatter plot of the coincidences at these four sites during both the LISTOS timeframe (Fig. 12a) and the longer 9-month period (Fig. 12b). During the LISTOS period the slope is 0.76, and a reasonably high *r*^2^ of 0.89 is caused by the large range of TrVCs observed at BayonneNJ and QueensNY. These results are similar to those at all nine locations during the LISTOS timeframe (Fig. 10a) with the same median percent difference. The number of coincidences through the LISTOS months is low (*n* = 58) due to the Δ_CS_ threshold being frequently exceeded (Table 7). The number and dynamic range of observations is greater when extended through the rest of the year (*n* = 195). The overall median percent difference is 8% lower over the 9-month period (−27%) than the LISTOS timeframe (−19%), and though it is not visually apparent in Fig. 11e, this drop is reflected by a decrease in the median percent difference at QueensNY (Table 6). At QueensNY, the median percent difference for TrVCs becomes more negative at higher magnitudes of TrVC; Pandora TrVCs less than/greater than 15×10^15^ molecules cm^−2^ have a median percent difference of −15% and −33%, respectively, at this site. Despite large day-to-day variations and changes in dynamic range through the seasons, the linear statistics for the aggregated data at these four sites are largely unchanged when comparing the LISTOS time frame to the extended 9-month period (2.5% difference in slope and 0.01 range in *r*^2^).

## 6 Overall evaluation of TROPOMI v1.2 NO_2_ TrVCs

Tables 5 and 7 summarize the overall results of TROPOMI TrVC comparisons to the airborne and Pandora spectrometers from this work. No matter the reference dataset or data selection criteria, linear regression and percent difference statistics indicate that in this urban coastal region the v1.2 TROPOMI standard TrVC product has a low bias. Median TROPOMI NO_2_ TrVCs are 19% and 33% lower than airborne and Pandora TrVCs, respectively, during the LISTOS timeframe. These different values are partially related to the characteristics of sampling at different TrVC ranges between the two datasets. One-third (130) of the airborne coincidences have TrVC less than 3×10^15^ molecules cm^−2^, with no observed bias between the two measurements, while only 19 of the 156 Pandora coincidences have TrVC less than 3×10^15^ molecules cm^−2^, with TROPOMI having a low bias of −21% at these cleanest levels. At higher TrVC magnitudes (greater than 6×10^15^ molecules cm^−2^), the percent differences of TROPOMI from aircraft (−29%) and Pandora (−31%) are more similar to each other. Lesser polluted columns are more sensitive to uncertainties related to the stratospheric columns, references, and other assumptions (which are different between all retrievals), whereas at more polluted levels the bias is more attributed to uncertainties in tropospheric air mass factors.

Overall these results are consistent with other studies using independent measurements to evaluate the TROPOMI NO_2_ products, as they also found that the TROPOMI NO_2_ product has a low bias in the Canadian Oil Sands (Griffin et al., 2019); Toronto, Canada (Zhao et al., 2020); Paris, France (Lorente et al., 2019); polluted scenes (> 10×10^15^ molecules cm^−2^) near Helsinki (Ialongo et al., 2020); Brussels, Belgium (Dimitropoulou et al., 2020); China (Liu et al., 2020); Munich, Germany (Chan et al., 2020); and Belgium (Tack et al., 2020). Verhoelst et al. (2020) completed a comprehensive analysis of TROPOMI NO_2_ products using broad networks of Pandora direct-sun and MAX-DOAS observations and also saw a low bias in the tropospheric product, including consistent results with three Pandora spectrometers used in this analysis (QueensNY, BronxNY, and BayonneNJ) with similar patterns in results (e.g., BronxNY, QueensNY, and BayonneNJ having a median percent difference of −15%, −23%, −41% (this work) vs. −13%, −26%, and −31% (Verhoelst et al., 2020), respectively). Slight differences are expected due to different date windows and coincidence criteria. Tack et al. (2020) also evaluate TROPOMI NO_2_ using an airborne spectrometer, and they reported a −14% bias in the TROPOMI standard product vs. airborne measurements collected over urban areas in Belgium in 2019. Many of these studies found improvement by using higher-resolution regional model a priori profile shapes in the AMF calculation for TROPOMI. In this study, recalculating the TROPOMI tropospheric AMF with the higher-resolution 12 km NAMCMAQ analysis resolves some of the low bias in TROPOMI TrVCs, improving median percent differences from −19% to −7% with respect to airborne data and from −33% to −19% with respect to Pandora data. However, despite this improvement, there is still a persistent low bias in the TROPOMI TrVCs. This contrasts from the results of the Tack et al. (2020) study that found that the bias improved to −1% when recalculating AMFs with a 0.1° spatial resolution from a CAMS regional ensemble. Though differences could be due to region-specific biases (NYC vs. Belgium), airborne retrieval biases, or different filtering techniques, such as the Δ_CS_ filter.

This analysis is impacted by influences of cloud pressure in the TROPOMI retrieval. Invoking the Δ_CS_ criterion increases (worsens) the overall TROPOMI low bias as it removes a high bias caused by assumed cloud shielding in the AMF calculation in cloud-free scenes. In all comparisons shown in Tables 5 and 7, the median percent difference is more negative (worse) when only points with Δ_CS_ less than 50hPa are included, and the effect is more pronounced for TROPOMI-NAMCMAQ coincidences (decreasing 10%–11%) than for TROPOMI standard (decreasing 4%–8%). Invoking the criterion also consistently improves the correlation in every case by removing many of the outlier points, as intended. The most striking examples are the airborne comparison with TROPOMI-NAMCMAQ (*r*^2^ improved from 0.83 to 0.95) and Pandora comparison with TROPOMI standard for the four-site subset of the LISTOS period (*r*^2^ improved from 0.79 to 0.88).

## 7 Conclusions

The operational nature of the S5P TROPOMI mission as part of the Copernicus program marks an important step forward in monitoring of the environment, amplifying the need for increased validation capacity of satellite trace gas data. The datasets collected in support of the Long Island Sound Tropospheric Ozone Study during summer 2018 and as part of the PAMS-EM program are exceptional for evaluation of TROPOMI TrVCs, providing a robust set of independent remotely sensed NO_2_ column densities from airborne spectrometers (13 mapping flights from 25 June 2018 to 6 September 2018) and a network of nine ground-based Pandora spectrometer systems.

Previous studies have shown that Pandora direct-sun NO_2_ columns are valuable for validating airborne spectrometer retrievals due to their high precision and temporal resolution and comparable spatial resolution (e.g., Nowlan et al., 2016; Judd et al., 2019). In this study, the airborne spectrometer data are highly correlated with Pandora measurements with a slope of 1.03, an offset of −0.4×10^15^ molecules cm^−2^, and *r*^2^ = 0.92. Much of the remaining scatter in the data can be attributed to the spatiotemporal heterogeneity of NO_2_ in this urban coastal environment, as evaluating only the less temporally varying measurements shows similar statistics but a higher *r*^2^ of 0.96. Though singular comparisons can exceed differences of 25%, overall the majority of the coincidences fall well within ±25% and 81% of the coincidences fall within the reported accuracy of Pandora of 2.69×10^15^ molecules cm^−2^. These results give confidence for using both datasets to assess the TROPOMI TrVC product.

The combination of these two reference measurements in one region presents unique strengths for validation of TROPOMI TrVCs over a domain with large variations in NO_2_. Pandora measurements are useful for evaluating space-based and aircraft-based retrievals due to their ability to observe continuously in one location for long time periods. However, the impact of subpixel heterogeneity within satellite pixel areas can lead to mismatches between the Pandora and satellite observations despite the much-improved spatial resolution of TROPOMI. Airborne spectrometers are typically only deployed for short periods of time, but their observations are more spatially representative of the satellite measurements with the added capability of retrieving at subpixel resolutions over the entire TROPOMI pixel areas they overfly. In this study, the strengths of the two reference measurements were able to be combined. TROPOMI comparisons to airborne TrVCs are more correlated than Pandora comparisons during the LISTOS timeframe (*r*^2^ = 0.96 vs. 0.84). Additionally, the long-term deployment of Pandora instruments as part of the PAMS-EM program allowed TROPOMI TrVCs to be assessed over multiple seasons. We find the strongest impact of seasonality is the extension of the TrVC dynamic range sampled in the winter months, providing more robust statistical fits though not very significant changes in the statistics overall between the two time periods.

During the LISTOS timeframe, TROPOMI standard TrVC data have a low bias in comparison to Pandora and airborne TrVCs of −33% and −19%, respectively. This bias improves to −19% and −7% when TROPOMI TrVCs are recalculated using AMFs with the 12 km NAMCMAQ a priori profile. These results are obtained by screening out cases where cloud shielding estimated in the TROPOMI retrieval occurred over cloud-free scenes, which tend to compensate partially for the TROPOMI TrVC low bias and introduce significant artifacts that degrade correlations with reference measurements. These instances of shielding were found where the 0.5°×0.5° surface reflectivity climatology used as a priori in the AMF calculation was insufficient in resolution to capture bright urban surfaces. This results in a positive cloud radiative fraction but appears to only result in an outlier when these scenes also have errors in the cloud pressure assuming shielding in cloud-free scenes. Future exploration of cloud-based coincidence criteria would help in identifying effects of cloud parameters and surface reflectivity on NO_2_ trace gas comparisons as well as other evaluations of near-surface weighted trace gases such as HCHO. It will also help in evaluating how these sensitivities change as cloud retrievals, surface reflectivity input, and their implementation into the trace gas retrievals evolve in future versions (e.g., in v1.3, implemented after 19 March 2019, the FRESCO-S cloud retrieval was updated to adjust surface albedo in cloud-free areas where the surface albedo climatology is too low, as discussed in Eskes and Eichmann, 2019).

We find the v1.2 TROPOMI standard TrVCs to be within the validation requirements for the mission (bias within ±25%–50%; van Geffen et al., 2019) but with a persistent low bias in the NYC region. While some of the bias is removed by the incorporation of a higher-resolution a priori vertical profile, there is still a low bias in the TROPOMI NO_2_ TrVC retrieval, which indicates the need for improved a priori assumptions in the AMF calculations. This analysis looked at the impacts of a priori NO_2_ profiles at a moderately higher resolution and of clouds, and future work should also explore effects of surface reflectivity. A component not explicitly explored in this work, which could be in the future, is the potential impact of aerosols on the TROPOMI retrieval and whether their indirect accounting through the cloud retrieval accurately reflects the impacts within the radiative transfer calculations for the air mass factor calculation (e.g., Leitão et al., 2010; Ma et al., 2013; Jin et al., 2016). Some differences between TROPOMI and airborne TrVCs can be related to differences in a priori assumptions between the TROPOMI and airborne retrievals; Lorente et al. (2017) discussed that the structural uncertainty in tropospheric air mass factors is up to 42% in polluted regions due to different retrieval methodologies. Future comparisons should consider using common methodologies for AMF calculation for both airborne and TROPOMI TrVCs to better quantify the sensitivity of specific a priori assumptions in AMF calculations.

As the spatial and temporal resolution of satellite-based observations have and will continue to improve in the near future, gathering large datasets of coincident observations with airborne spectrometers becomes more feasible during air quality field studies. This provides a unique perspective for satellite validation and evaluation strategies, especially with the added information on subpixel variability compared to traditional reference datasets. The datasets presented in this work and others like it will continue to provide a reference for validating and evaluating UV–VIS trace gas retrievals, including the assessment of reprocessed TROPOMI products and near-future geostationary measurements.

## Supplementary Material

Supplement1

## Figures and Tables

**Table 1.** A priori input for tropospheric AMF calculations for TROPOMI and airborne TrVCs.

	TROPOMI v1.2	Airborne
A priori NO2 profile shape	TM5-MP 1^◦^ ×1^◦^ model (Williams et al., 2017)	Troposphere: 12km NAMCMAQ (Stajner et al., 2011) Stratosphere: PRATMO climatology (Prather, 1992; McLinden et al., 2000) bias corrected daily with TROPOMI stratospheric vertical columns
Surface reflectivity	OMI 0.5^◦^ ×0.5^◦^ 5-year climatology (Kleipool et al., 2008)	Land: MCD43A1 daily L3 500m v006 product (Lucht et al., 2000; Schaaf and Wang, 2015) averaged over the period of the campaign Water: assumed Lambertian reflectance of at least 3% and Cox–Munk kernel
Pressure/temperature profiles	TM5-MP 1^◦^ ×1^◦^ model driven by the ECMWF corrected with a 3km DEM	Troposphere: 12km NAMCMAQ (Stajner et al., 2011) Stratosphere: 1^◦^ RAQMS (Pierce et al., 2009)
Clouds	FRESCO-S (Loyola et al., 2018)	Cloudy scenes are not included in this analysis

**Table 2.** Comparison of GeoTASO and GCAS.

	GeoTASO	GCAS
Spectral range	290–390nm, 415–695nm	300–490nm, 480–900nm
Spectral resolution	0.43nm, 0.88nm	0.6nm, 2.8nm
Size/weight	90kg	36kg
Detector dimensions	1056 spectral×1033 spatial	1072 spectral×1024 spatial
Integration times	250ms	225 to 750ms

Native spatial resolution	Approximately 250m×250m	
Field of view	45°	

References	Leitch et al. (2014)	Kowalewski and Janz (2014)
	Nowlan et al. (2016)	Nowlan et al. (2018)
	Judd et al. (2019)	

**Table 3.** GeoTASO/GCAS flight summary for LISTOS. Flights with shaded boxes are not considered in this analysis.

Flight	Date	Time (UTC fractional hour)	Pollution scale (95th percentile × 10^15^ molecules cm^−2^)	% Cloudy pixels	No. of valid Pandora coincidences	No. of valid TROPOMI coincidences	Flight pattern type (Fig. 1)
1	18 Jun 2018	12.0–15.6					Large
2		17.0–20.7					Large

3	25 Jun 2018	12.5–15.7	7.3	10	5	34	Small
4		16.8–20.3	7.2	5			Small

5	30 Jun 2018	12.2–15.6	11.2	0	9	65	Small
6		16.7–20.4	13.5	1			Small

7	2 Jul 2018	11.4–16.6	14.5	0	7	18	Small
8		17.9–21.5	18.9	0			Small

9	19 Jul 2018	11.4–15.3	17.9	0	11	47	Large
10		16.9–20.9	32.4	0			Large

11	20 Jul 2018	11.4–15.3	30.4	3	15	38	Large
12		17.1–21.1	16.3	5			Large

13	5 Aug 2018	12.5–16.5	15.5	1	15	0	Large
14		17.8–22.3	10.2	5			Large

15	6 Aug 2018	11.7–16.0	21.3	0	13	11	Large
16		17.2–21.5	16.1	5			Small

17	15 Aug 2018	11.2–15.5	12.4	0	17	52	Large
18		17.0–21.6	9.8	5			Large

19	16 Aug 2018	11.3–15.3	13.7	17	16	31	Small
20		17.3–21.5	9.8	2			Small

21	24 Aug 2018	10.9–15.3	14.7	0	18	32	Large
22		16.6–21.0	37.8	4			Large

23	28 Aug 2018	11.3–15.3	16.6	0	15	10	Small
24		16.6–20.3	16.0	2			Small

25	29 Aug 2018	11.2–15.1	16.8	0	17	17	Small
26		16.6–20.8	14.0	3			Small

27	6 Sep 2018	11.9–15.8	11.8	9	13	33	Small
28		17.2–21.4	12.2	5			Small

29	3 Oct 2018	12.3–16.7					Small
30		18.2–21.8					Small

31	19 Oct 2018	12.8–15.2					Small
32		16.8–20.3					Small

**Table 4.** Pandora sites and time of operation. Shaded boxes represent the months of LISTOS.

Pandora name	Latitude, longitude		Months with valid data (number of measurement days per month)									

			2018						2019			

			J	J	A	S	O	N	D	J	F	M
QueensNY	40.7361,	−73.8215	5	23	27	26	27	27	25	26	26	29
BronxNY	40.8679,	−73.8781	6	29	29	16	21	10	-	-	-	-
BayonneNJ	40.6703,	−74.1261	-	21	31	27	26	25	25	26	24	28
FlaxPondNY	40.9635,	−73.1402	2	13	28	19	5	-	-	-	-	-
WestportCT	41.1183,	−73.3367	5	19	29	25	27	24	26	23	5	22
NewHavenCT	41.3014,	−72.9029	6	30	29	19	19	14	24	15	-	-
RutgersNJ	40.4622,	−74.4294	2	30	30	21	27	22	25	21	5	21
MadisonCT	41.2568,	−72.5533	7	13	-	-	-	-	-	-	-	-
BranfordCT	41.2420,	−72.7604	-	9	30	4	-	-	-	-	-	-

**Table 5.** Statistics for TROPOMI and airborne comparisons with the coincidence criteria of CRF<50% and aircraft sampled within ±30 min of the S5P overpass with different a priori profiles and indication of whether the Δ_CS_ threshold is applied.

TROPOMI dataset	ΔCS <50hPa	RMA fit	*r*^2^	Median percent difference	*N*
Standard slant column	No	y = 0.58×+1.5×10^15^	0.95	−12%	621
	Yes	y = 0.59×+1.5×10^15^	0.96	−13%	388

Standard TrVC	No	y = 0.71×+0.9×10^15^	0.90	−11%	621
	Yes	y = 0.68×+0.6×10^15^	0.96	−19%	388

NAMCMAQ TrVC	No	y = 0.84×+1.0×10^15^	0.83	4%	621
	Yes	y = 0.77×+0.7×10^15^	0.95	−7%	388

**Table 6.** Statistics between Pandora and TROPOMI by site for the LISTOS period as well as extended to 19 March 2019.

LISTOS only (June-September 2018)						Valid data from June 2018 to March 2019				

Site	RMA fit	*r*^2^	median % difference	median column difference	*N*	RMA fit	*r*^2^	median % difference	median column difference	*N*
QueensNY	*Y* = 0:77× +0:6 × 10^15^	0.87	−9%	−0:5 × 10^15^	22	*Y* = 0:63× +1:3 × 10^15^	0.76	−23%	−2:1 × 10^15^	68
BronxNY	*Y* = 0.81× +0.03 × 10^15^	0.90	−15%	−1:1 × 10^15^	20	*Y* = 0.73× +0.5 × 10^15^	0.87	−15%	−1:1 × 10^15^	33
BayonneNJ	*Y* = 0.84× −2.1 × 10^15^	0.87	−38%	−4:1 × 10^15^	9	*Y* = 0.74× −1.8 × 10^15^	0.88	−41%	−5:3 × 10^15^	45
BayonneNJ	*Y* = 0.84× +2.1 × 10^15^	0.87	−38%	−4:1 × 10^15^	9	*Y* = 0.74× +1.8 × 10^15^	0.88	−41%	−5:3 × 10^15^	45
RutgersNJ	*Y* = 0.63× +0.4 × 10^15^	0.69	−26%	−0:9 × 10^15^	6	*Y* = 0.76× −0.1 × 10^15^	0.95	−24%	−1:4 × 10^15^	33
FlaxPondNY	*Y* = 0.53× +0.4 × 10^15^	0.59	−37%	−1:7 × 10^15^	23	*Y* = 0.53× +0.5 × 10^15^	0.60	−37%	−1:4 × 10^15^	25
NewHavenCT	*Y* = 0.52× −0.5 × 10^15^	0.29	−52%	−2:7 × 10^15^	25	*Y* = 0.70× −1.3 × 10^15^	0.71	−50%	−2:7 × 10^15^	47
BranfordCT	*Y* = 1.22× −2.7 × 10^15^	0.31	−46%	−1:9 × 10^15^	22	*Y* = 1.2× −2.7 × 10^15^	0.31	−46%	−1:9 × 10^15^	22
MadisonCT	*Y* = 1.94× −2.7 × 10^15^	0.12	−24%	−0:6 × 10^15^	8	*Y* = 2.4× −3.9 × 10^15^	0.02	−24%	−0:7 × 10^15^	11

**Table 7.** Summary statistics for Pandora and TROPOMI over the LISTOS time period and extended to 19 March 2019 with different a priori profiles and indication of whether the ΔCS threshold is applied.

Time period	Location	TROPOMI dataset	ΔCS <50hPa	RMA fit	*r*^2^	Median percent difference	*N*
LISTOS only	All sites	Standard	No	*y* =0.82×−0.6×10^15^	0.79	−30%	294
			Yes	*y* =0.80×−0.7×10^15^	0.84	−33%	156
		
		NAMCMAQ	No	*y* =1.05×−0.7×10^15^	0.77	−9%	294
			Yes	*y* =0.82×−0.2×10^15^	0.80	−19%	156

LISTOS only	RutgersNJ		No	*y* =0.78×−0.5×10^15^	0.79	−17%	132
	BayonneNJ	Standard	Yes	*y* =0.76×+0.1×10^15^	0.88	−19%	58
			
26 June 2018–	QueensNY		No	*y* =0.74×+0.2×10^15^	0.82	−21%	373
19 March 2019	WestportCT		Yes	*y* =0.78×−0.3×10^15^	0.87	−27%	195
