# Supplementary material for: Evaluating Sentinel-5P TROPOMI tropospheric NO2 column densities with airborne and Pandora spectrometers near New York City and Long Island Sound
Source: Atmos Meas Tech. Author manuscript; Available in PMC 2021 Nov 17. (PMC8193800; doi:10.5194/amt-13-6113-2020)
Supplement: Supplement1 [file NIHMS1687816-supplement-Supplement1.pdf]

Supplement of Atmos. Meas. Tech., 13, 6113–6140, 2020
https://doi.org/10.5194/amt-13-6113-2020-supplement
© Author(s) 2020. This work is distributed under
the Creative Commons Attribution 4.0 License.

Atmospheric Measurement Techniques
Open Access

Supplement of

Evaluating Sentinel-5P TROPOMI tropospheric NO_2 column densities with airborne and Pandora spectrometers near New York City and Long Island Sound

Laura M. Judd et al.

Correspondence to: Laura M. Judd (laura.m.judd@nasa.gov)

The copyright of individual parts of the supplement might differ from the CC BY 4.0 License.

S1. Sensitivity of Airborne/Pandora comparisons to coincidence criteria

Table S1 shows the sensitivity of the aircraft-Pandora aggregate linear regression to the coincidence criteria. Coincidence criteria varied to include the radial distance from the Pandora location, Pandora viewing azimuth, temporal window of Pandora data, temporal averaging and filtering of Pandora data, and the range in the S5P TROPOMI overpass time.

Table S1: Airborne v. Pandora column statistics based on temporal and spatial coincidence criteria.

	Coincidence Criteria		Statistics			
**Row**	**Temporal Criteria**	**Spatial Aircraft Criteria**	**Slope**	**Offset (molecules cm^{-2})**	**r^2**	**N**
A	Temporally closest Pandora coincidence	Median 250 m radius	1.13±0.03	$-0.8\times10^{15}\pm 0.3\times10^{15}$	0.91	129
B		Median 750 m radius	1.03±0.03	$-0.4\times10^{15}\pm 0.2\times10^{15}$	0.92	171
C		Median 1.5 km radius	0.98±0.04	$-0.1\times10^{15}\pm 0.3\times10^{15}$	0.89	177
D		Median 750 m radius (±45 degree sector)	1.07±0.03	$-0.6\times10^{15}\pm 0.3\times10^{15}$	0.92	169
E		Median 750 m radius (±22.5 degree sector)	1.08±0.03	$-0.7\times10^{15}\pm 0.3\times10^{15}$	0.92	167
F	Pandora Median ±5 min	Median 750m radius	1.03±0.03	$-0.4\times10^{15}\pm 0.3\times10^{15}$	0.91	171
G	Pandora Median ±15 min		1.03±0.03	$0.3\times10^{15}\pm 0.2\times10^{15}$	0.92	176
H	Temporally closest Pandora coincidence but excluding Temporally variable data		1.05±0.04	$-0.3\times10^{15}\pm 0.3\times10^{15}$	0.96	97
I	Coincidences during the TROPOMI Overpass Window (16.7-19.0 UTC)		1.14±0.03	$-1.1\ \mathrm{x}10^{15}\pm 0.3\times10^{15}$	0.94	47

Row B presents results with the baseline criteria used in this analysis: the instantaneous Pandora observation closest in time to the aircraft overflight within ±5 minutes of the overflight and the median of the airborne data within a 750 m radius of the sites. These were the same criteria implemented in Judd et al. (2019). This is the dataset that results in the highest r^2 except for the subsets based on temporal variability and TROPOMI temporal window (Rows H and I)

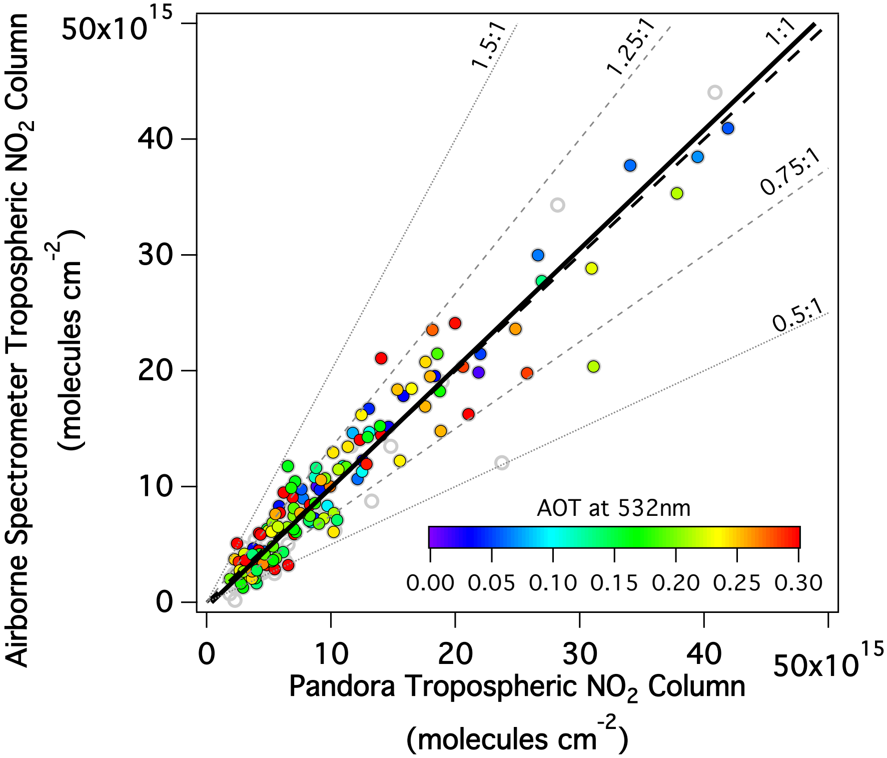

Figure S1: Scatter plot showing the temporally closest Pandora TrVC to the aircraft overpass vs. the median airborne TrVC within a 750 m radius of the Pandora site (same as Figure 3) colored by AOT measured by the HALO Lidar at 532 nm. Open grey circles are coincidences that occurred without HALO data.

Figure S1 shows the impact of aerosol loading on the Pandora and airborne TrVC comparison as aerosols are not included as a priori input in the airborne AMF calculation. Over 90% of these coincidences have an AOT at 532 nm < 0.3, two coincidences are above 0.5 with a max of 0.7. There does not appear to be any relation to aerosol loading in the Pandora/aircraft comparison, though future work could calculate AMFs explicitly accounting for aerosol profile properties and loading for times when HALO data is available.

S2. Sensitivity of Airborne/TROPOMI comparisons to coincidence criteria

Table S2 shows the sensitivity of coincidence criteria on airborne TrVC comparisons to TROPOMI. Coincidence criteria varied to include the temporal window in which aircraft data is extracted from the TROPOMI overpass time, the percentage of the TROPOMI pixel that is mapped by the aircraft within that temporal window, cloud radiative fraction (CRF), and Δ_{CS}, which is defined as the difference in surface and cloud pressures in the TROPOMI product file, discussed in Sect 4.1 in the main manuscript. The applied coincidence criteria in this work is shown in Row M; these criteria are airborne data collected within ± 30 min of the TROPOMI overpass for pixels that are at least 75% mapped with CRFs less than 50% and $\Delta_{CS} < 50$

hPa. This set of criteria resulted in nearly the highest r^2, where the exception was for all the same critieria but for CRFs confined to less than 20-30%. However, the tradeoff for allowing CRFs up to 50% results in more data points included in the analysis with no change in the slope.

Table S2: Airborne vs. TROPOMI statistics for varying temporal windows, mapped percentage by the aircraft of TROPOMI pixels, Cloud Radiative Fraction (CRF), Δ_{CS} filter.

Row	Temporal Window	% Mapped	CRF	$\Delta_{CS} < 50$ hPa Filter	Slope	Intercept ×10[15]	r^2	N
A	**± 60 min**	**75%**			0.67±0.03	1.0±0.2	0.80	1068
B		**75%**			0.71±0.02	0.9±0.1	0.90	621
C	**± 30 min**	**50%**	**< 50%**	No	0.72±0.02	1.0±0.1	0.89	814
D		**25%**			0.75±0.03	1.1±0.1	0.83	1004
E	**± 15 min**	**75%**			0.72±0.03	0.8±0.2	0.93	285
F			**< 50%**		0.71±0.02	0.9±0.1	0.90	621
G			**< 30%**	No	0.70±0.03	0.9±0.1	0.94	452
H			**< 20%**		0.70±0.02	0.8±0.1	0.96	290
I			**< 10%**		0.67±0.02	0.9±0.1	0.95	165
J		**75%**	**< 50%**		0.68±0.01	0.6±0.1	0.96	388
K	**± 30 min**		**< 30%**		0.68±0.01	0.8±0.1	0.97	313
L			**< 20%**	Yes	0.68±0.01	0.8±0.1	0.97	202
M			**< 10%**		0.66±0.02	0.9±0.1	0.96	118
N		**50%**	**<50%**		0.70±0.01	0.7±0.1	0.93	487
O		**25%**			0.73±0.03	0.9±0.1	0.86	584

S3. Case Study Illustrating Sensitivity to Cloud Pressure for Δ_{CS} threshold

When comparing the aircraft data to TROPOMI, two outliers (apparent in Figures 6 and 7 in the main manuscript) extend well above the main population of data. These points occured on July 19th, 2018, for TROPOMI pixels viewing the urban areas of eastern New Jersey just across the river from Manhattan Island NYC (red outlined points in Figure S2). VIIRS true-color imagery (Figure 5 in the main manuscript) shows that there are zero clouds in the domain. Even without the presence of clouds, TROPOMI CRFs extend up to 33% over the LISTOS domain demonstrating how limiting CRFs to those below 20% only would exclude many comparisons over the bright urban surfaces in this domain.

For the outlier pixels, TROPOMI retrieves a 19 and 23% CRF, similar to nearby pixels which do not result in outliers. The difference lies within the retrieved cloud pressure in relation to the surface pressure. In the case of the two outliers, the cloud pressure is above 800 hPa, which is reflected in the loss of sensitivity as seen in the box AMF profiles in grey in Figure S2. The impact of aerosols has been ruled out for this instance as the airborne HALO instrument retrieved AOT at 532nm of 0.04. The other coincidences on this day have a median cloud pressure of 984 hPa with a standard deviation of 29 hPa (median surface pressure is 1016 hPa). The reported uncertainty in cloud pressure is 50 hPa in van Geffen et al., (2019).

Adjusting box AMFs to remove the estimated loss of sensitivity below the retrieved cloud level (red line in profiles in Figure S2) and recalculating tropospheric AMF results in an increase in AMF (changing from 0.61 and 0.68 to 1.00 and 1.09, respectively, using the 12 km NAMCMAQ a priori profile) which brings these two outliers into agreement with the rest of the data population (within the distribution of the red circles in Figure 7).

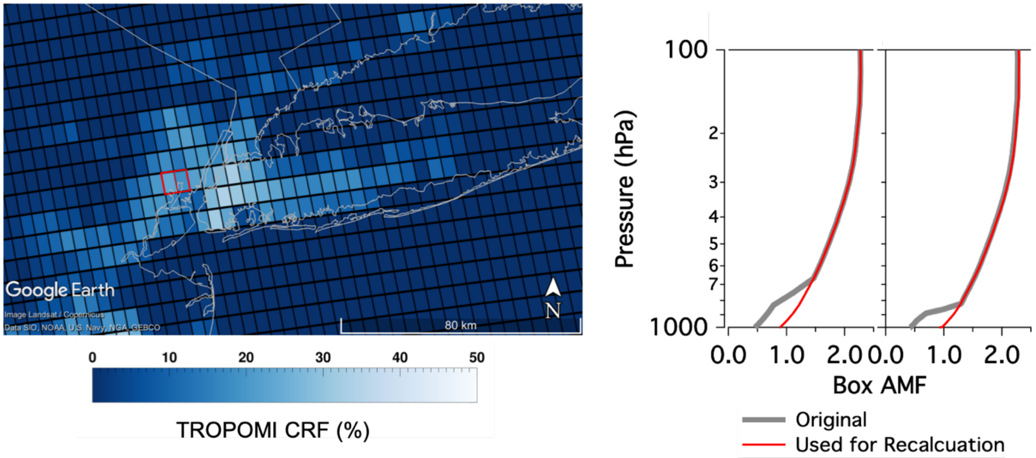

Figure S2: © Google Earth Map showing TROPOMI's CRF retrieved during the 19 July 2018 S5P overpass and original BoxAMFs (grey line) for the pixels outlined in red the map along with a second BoxAMF that is used to recalculate the TROPOMI tropospheric AMF with the removal of the impact of clouds

S4. Sensitivity of TROPOMI/Pandora comparisons to coincidence criteria

Table S3 shows the sensitivity of coincidence criteria on Pandora TrVC comparisons to TROPOMI. Coincidence criteria varied to include the temporal window in which Pandora data is analyzed from the TROPOMI overpass time, CRF, and the Δ_{CS} threshold discussed in Sect 4.1 in the main manuscript. The applied coincidence criteria are shown in Row C and Row J (repeated for ease of comparison in the table); these are the median Pandora TrVC within a ± 30 min window from the TROPOMI overpass with TROPOMI CRFs less than 50% and Δ_{CS} less than 50 hPA.

Comparison of Rows A, B, and D to Row C shows the sensitivity to length of time over which the median is calculated from Pandora data. Additionally, comparison of Row E to Rows A-D shows the effect of using the instantaneous Pandora observation closest in time to the TROPOMI overpass versus temporal averaging of the Pandora data. As the temporal window

gets smaller, the slope decreases likely due to spatial heteorgeneity in the TROPOMI sub-pixel area. The r^2 does not change when the temporal window is extended to ± 60 minutes, but ± 30 minutes is consistent with the airborne column comparisons which showed the effect of temporal mismatches beyond the ± 30 minute window. Additionally, comparing Rows F-I to Rows J-M shows the impact of applying the Δ_{CS} threshold. There is clear improvement in r^2 when applying the Δ_{CS} criterion.

Recent studies using Pandora data to evaluate TROPOMI NO_2 products have used different coincidence criteria in terms of the temporal window and statistics applied to Pandora data. Griffin et al. (2019) averaged Pandora data within ± 30 min window of the S5P overpass, similar to the current methodology but instead here the median is used to limit possible influence of isolated small-scale plumes that TROPOMI is likely not sensitive to. Ialongo et al. (2020) and Zhao et al. (2019) used a smaller temporal window of ± 10 minutes, though they used the average and closest coincidence, respectively. Unlike in airborne-Pandora comparisons, only considering Pandora coincidences where its TrVC does not vary more than 30% within a ± 30 min window does not improve r^2, but does show an increase in slope.

Table S3 TROPOMI v. Pandora column statistics based on coincidence criteria for the LISTOS time period

Coincidence Criteria			Statistics			
**Row**	**Temporal Window for Pandora Median**	**Cloud Criteria**	**Slope**	**Offset (×10^{15} molecules cm^{-2})**	**r^2**	**N**
A	± 90 min	Δ_{CS} < 50 hPa + CRF < 50%	0.85±0.05	-0.9±0.3	0.83	157
B	± 60 min		0.82±0.04	-0.8±0.2	0.84	156
C	± 30 min		0.80±0.04	-0.7±0.2	0.84	156
D	± 15 min		0.75±0.04	-0.5±0.2	0.82	151
E	Closest Coincidence		0.73±0.04	-0.4±0.2	0.82	151
F	± 30 min	CRF<50%	0.82±0.12	-0.6±0.6	0.79	294
G		CRF<30%	0.91±0.11	-1.2±0.4	0.72	186
H		CRF<20%	0.85±0.09	-0.9±0.3	0.77	122
I		CRF<10%	0.91±NaN	-1.0±0.6	0.49	65
J		Δ_{CS} < 50 hPa + CRF < 50%	0.80±0.04	-0.7±0.2	0.84	156
K		Δ_{CS} < 50 hPa + CRF < 30%	0.82±0.06	-0.9±0.2	0.78	131
L		Δ_{CS} < 50 hPa + CRF < 20%	0.81±0.06	-0.8±0.2	0.83	90
M		Δ_{CS} < 50 hPa + CRF < 10%	1.01±0.01	-1.3±0.5	0.68	47
N	± 30 min, but excluding data Temporally variable data	Δ_{CS} < 50 hPa + CRF < 50%	0.86±0.09	-0.7±0.3	0.75	75

References

van Geffen, J., Eskes, H., Boersma, F., Maasakkers, J. D. and Veefkind, J. P.: TROPOMI ATBD of the total and tropospheric NO2 data products. http://www.tropomi.eu/sites/default/files/files/publicS5P-KNMI-L2-0005-RP-ATBD_NO2_data_products-20190206_v140.pdf (Accessed 14 April 2020), 2019.

Griffin, D., Zhao, X., McLinden, C. A., Boersma, F., Bourassa, A., Dammers, E., Degenstein, D., Eskes, H., Fehr, L., Fioletov, V., Hayden, K., Kharol, S. K., Li, S.-M., Makar, P., Martin, R. V., Mihele, C., Mittermeier, R. L., Krotkov, N., Sneep, M., Lamsal, L. N., Linden, M. ter, Geffen, J. van, Veefkind, P. and Wolde, M.: High-Resolution Mapping of Nitrogen Dioxide With TROPOMI: First Results and Validation Over the Canadian Oil Sands, Geophysical Research Letters, 46(2), 1049–1060, doi:10.1029/2018GL081095, 2019.

Ialongo, I., Virta, H., Eskes, H., Hovila, J. and Douros, J.: Comparison of TROPOMI/Sentinel-5 Precursor NO2 observations with ground-based measurements in Helsinki, Atmospheric Measurement Techniques, 13(1), 205–218, doi:10.5194/amt-13-205-2020, 2020.

Judd, L. M., Al-Saadi, J. A., Janz, S. J., Kowalewski, M. G., Pierce, R. B., Szykman, J. J., Valin, L. C., Swap, R., Cede, A., Mueller, M., Tiefengraber, M., Abuhassan, N. and Williams, D.: Evaluating the impact of spatial resolution on tropospheric NO2 column comparisons within urban areas using high-resolution airborne data, Atmospheric Measurement Techniques, 12(11), 6091–6111, doi:https://doi.org/10.5194/amt-12-6091-2019, 2019.

Zhao, X., Griffin, D., Fioletov, V., McLinden, C., Cede, A., Tiefengraber, M., Müller, M., Bognar, K., Strong, K., Boersma, F., Eskes, H., Davies, J., Ogyu, A. and Lee, S. C.: Assessment of the quality of TROPOMI high-spatial-resolution NO2 data products, , doi:10.5194/amt-2019-416, 2019.
